# Supplementary material for: Atomic-scale regulation of anionic and cationic migration in alkali metal batteries
Source: Nat Commun. 2021 Jul 7;12:4184. doi: 10.1038/s41467-021-24399-9 (PMC8263716; doi:10.1038/s41467-021-24399-9)
Supplement: Supplementary file 1 — Supplementary Information [file 41467_2021_24399_MOESM1_ESM.pdf]

Supplementary Information

Atomic-scale regulation of anionic and cationic migration in alkali metal batteries

Pan Xiong[1,2,#], Fan Zhang[2,#], Xiuyun Zhang[3,#], Yifan Liu[1], Yunyan Wu[1], Shijian Wang[2], Javad Safaei[2], Bing Sun[2], Renzhi Ma[4], Zongwen Liu[5], Yoshio Bando[4], Takayoshi Sasaki[4], Xin Wang[1], Junwu Zhu[1*], Guoxiu Wang[2*]

[1]Key Laboratory for Soft Chemistry and Functional Materials of Ministry Education, Nanjing University of Science and Technology, Nanjing, 210094, China

[2]Centre for Clean Energy Technology, School of Mathematical and Physical Sciences, University of Technology Sydney, NSW 2007, Australia

[3]College of Physical Science and Technology, Yangzhou University, Yangzhou, 225002, China

[4]International Center for Materials Nanoarchitectonics (WPI-MANA), National Institute for Materials Science (NIMS), Namiki 1-1, Tsukuba, Ibaraki, 305-0044, Japan

[5]School of Chemical and Biomolecular Engineering, The University of Sydney, NSW 2006, Australia

[#]These authors contributed equally: Pan Xiong, Fan Zhang, Xiuyun Zhang

[*]e-mail: zhujw@njust.edu.cn; guoxiu.wang@uts.edu.au

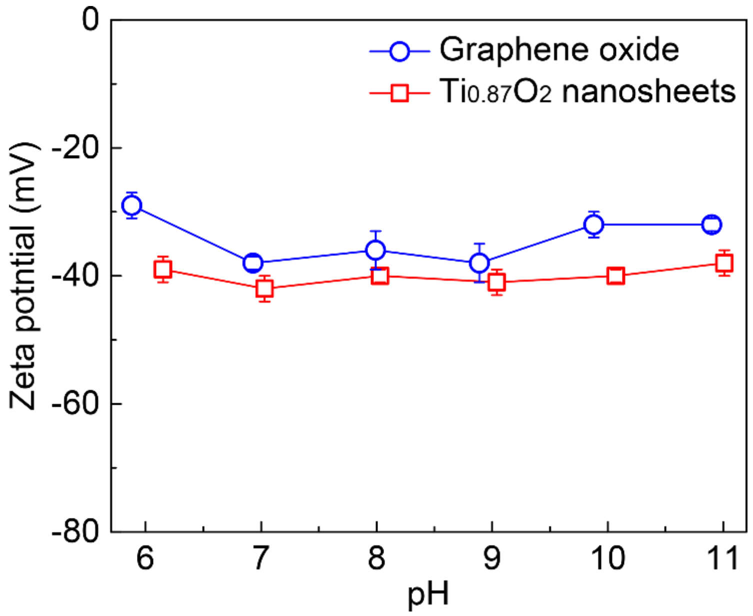

Supplementary Figure 1. Zeta-potentials of the suspensions of graphene oxide and $Ti_{0.87}O_2$ nanosheets.

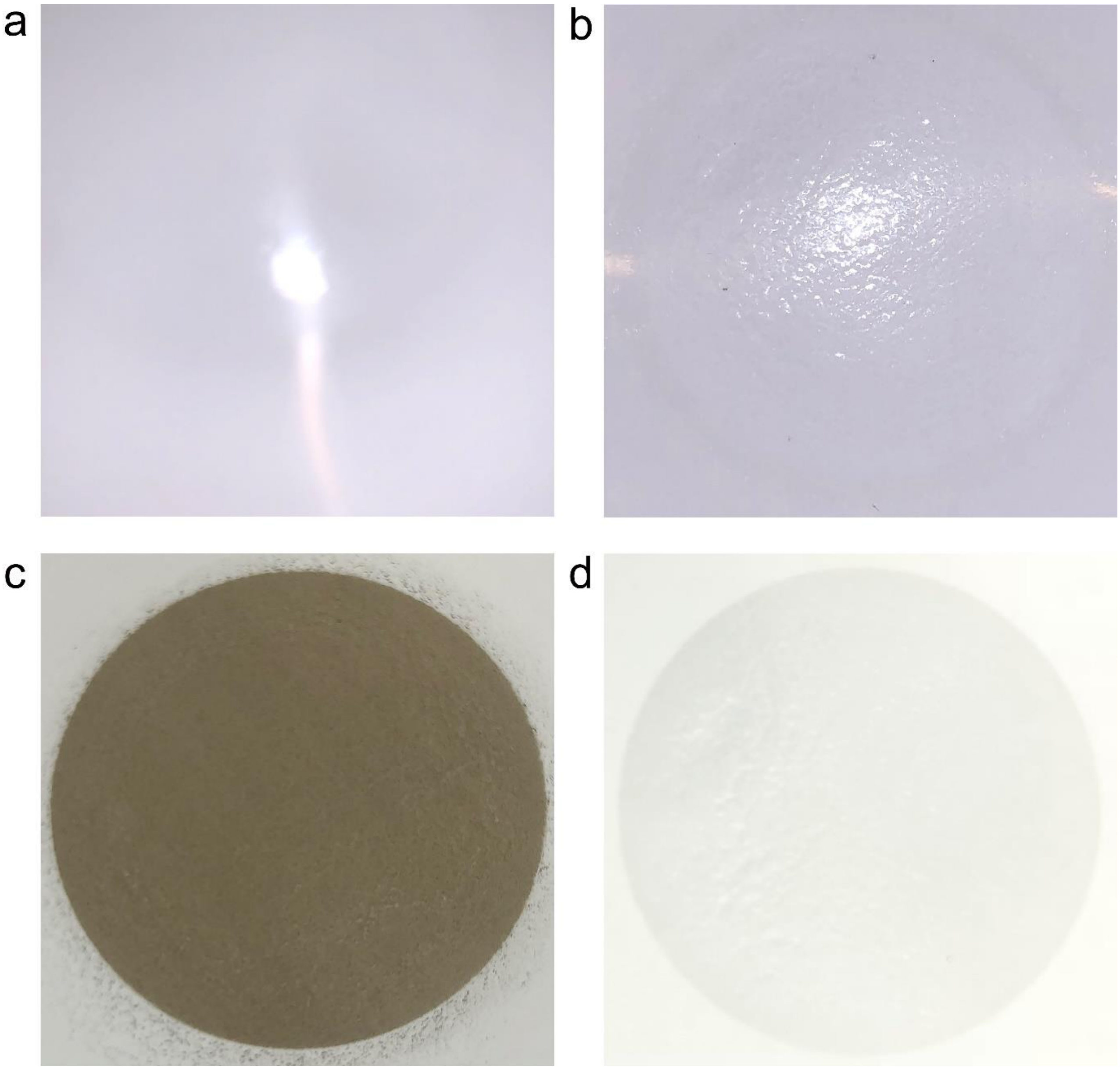

Supplementary Figure 2. Photographs of the (a) PP, (b) anatase TiO_2/PP, (c) GO/PP, and (d) $Ti_{0.87}O_2$/PP separators.

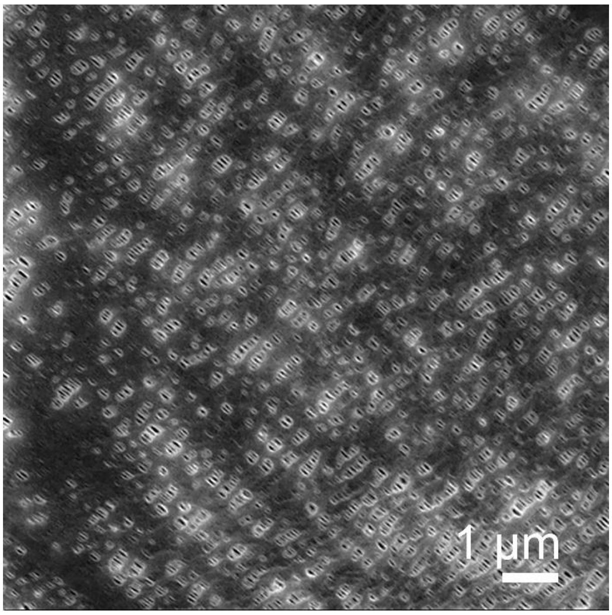

Supplementary Figure 3. SEM image of the commercial PP separators.

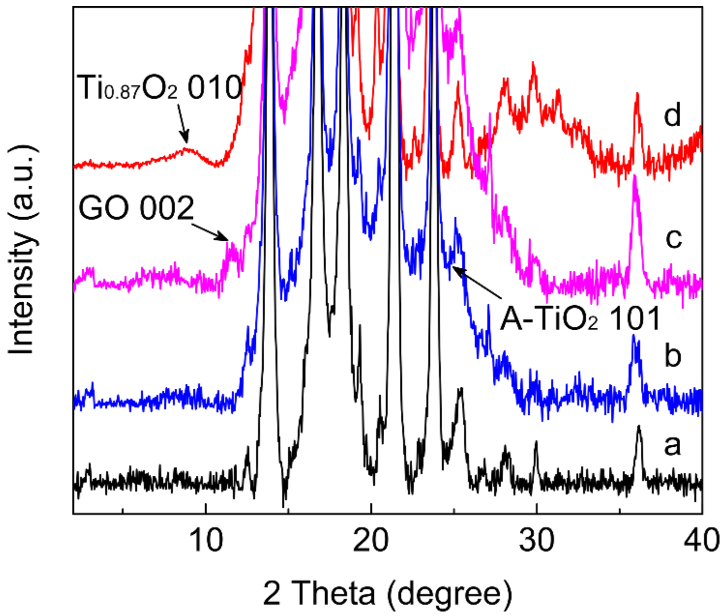

Supplementary Figure 4. XRD patterns of (a) PP, (b) anatase TiO_2/PP, (c) GO/PP, and (d) $Ti_{0.87}O_2$/PP separators. The 101 diffraction peak of anatase TiO_2 (A-TiO_2), 002 diffraction peak of GO and 010 diffraction peak of $Ti_{0.87}O_2$ were marked.

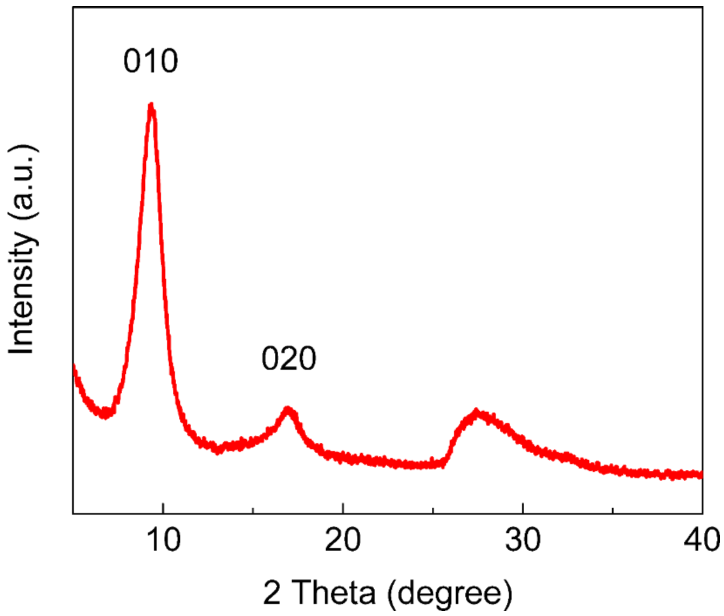

Supplementary Figure 5. XRD pattern for the $Ti_{0.87}O_2$ nanosheets without PP separators.

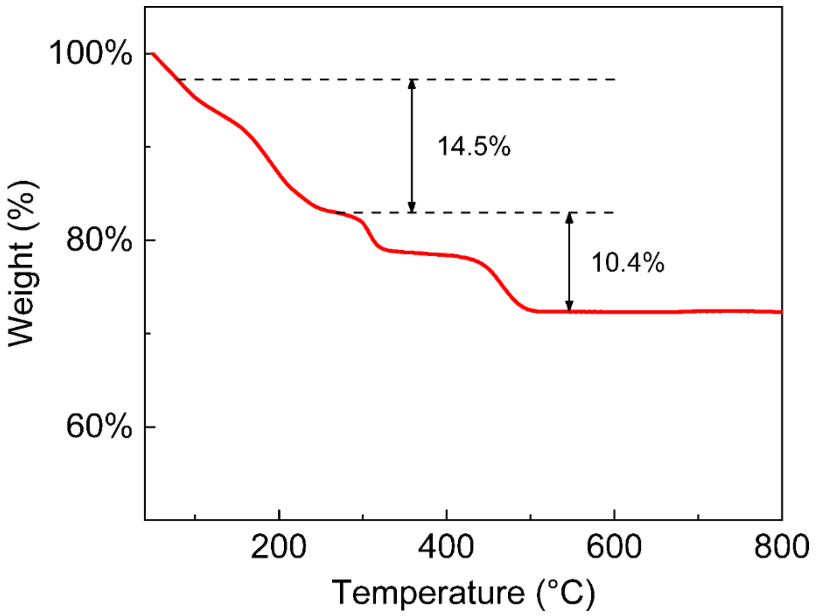

Supplementary Figure 6. Thermogravimetric curve for the nanosheet films without PP separators.

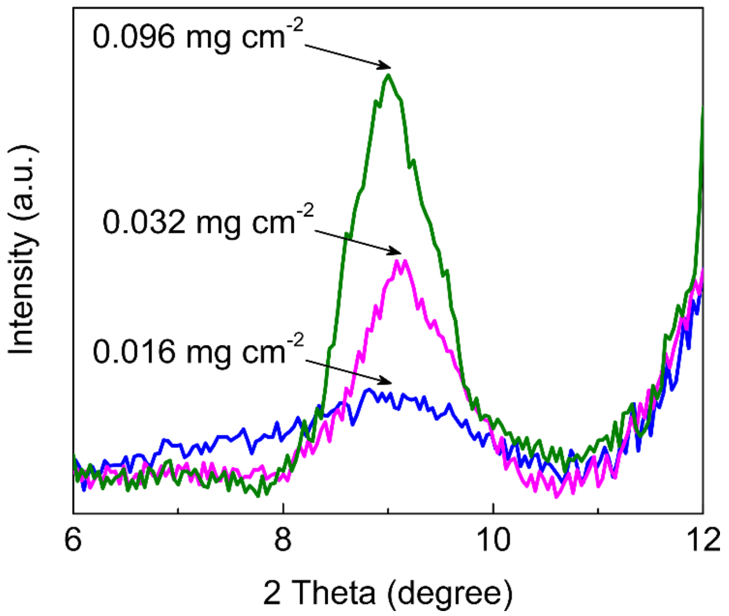

Supplementary Figure 7. XRD patterns of $Ti_{0.87}O_2$/PP separators with different surface area mass loadings.

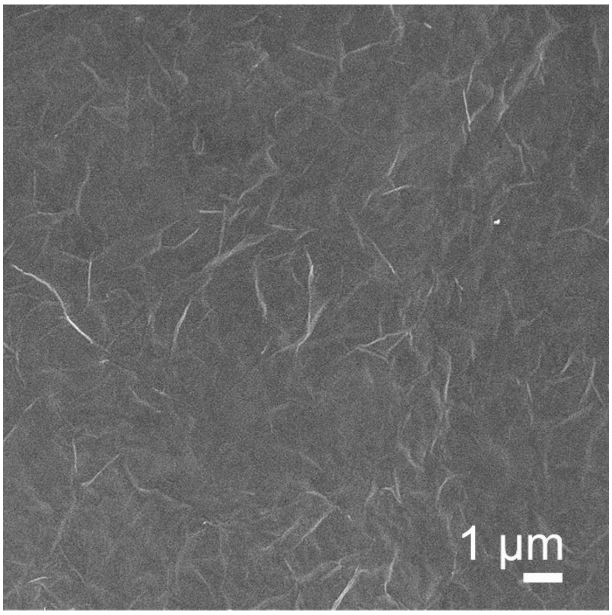

Supplementary Figure 8. SEM image of $Ti_{0.87}O_2$/PP separators with a surface area mass loading of 0.032 mg cm^{-2}.

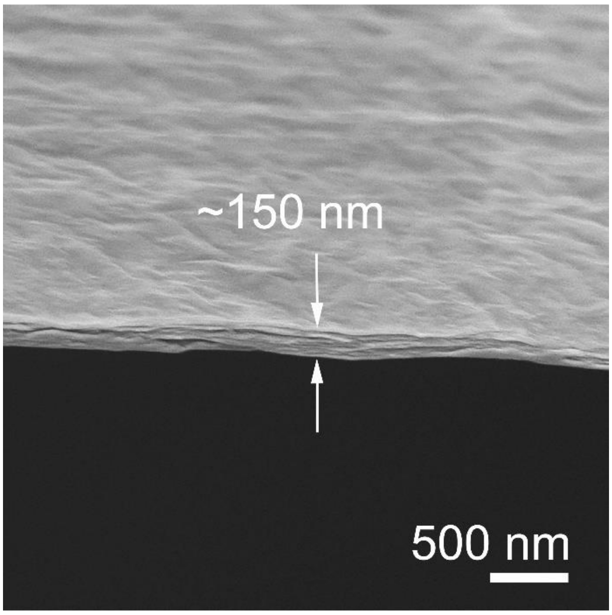

Supplementary Figure 9. Cross-section SEM image of $Ti_{0.87}O_2$/PP separators with a surface area mass loading of 0.032 mg cm^{-2}.

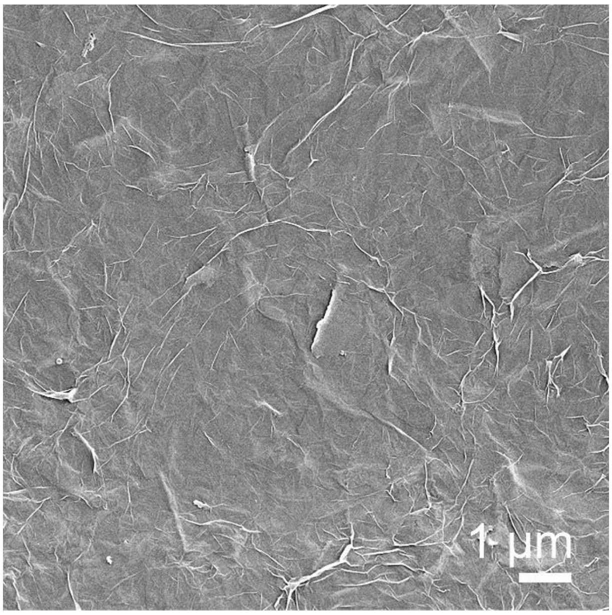

Supplementary Figure 10. SEM image of $Ti_{0.87}O_2$/PP separators with a surface area mass loading of 0.096 mg cm^{-2}.

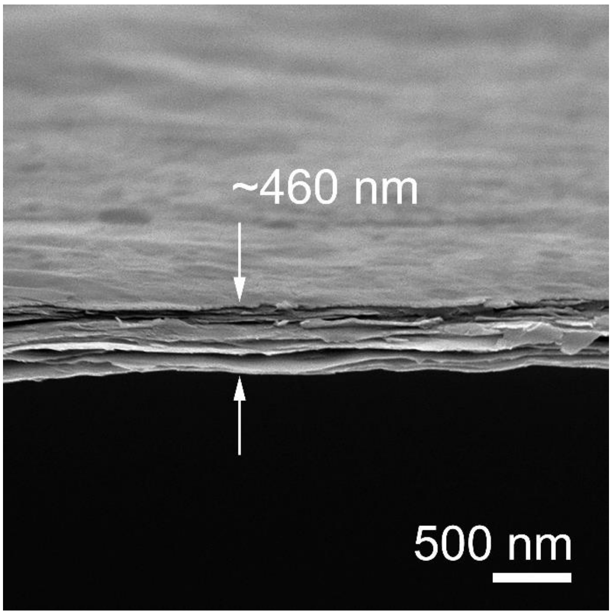

Supplementary Figure 11. Cross-section SEM image of $Ti_{0.87}O_2$/PP separators with a surface area mass loading of 0.096 mg cm^{-2}.

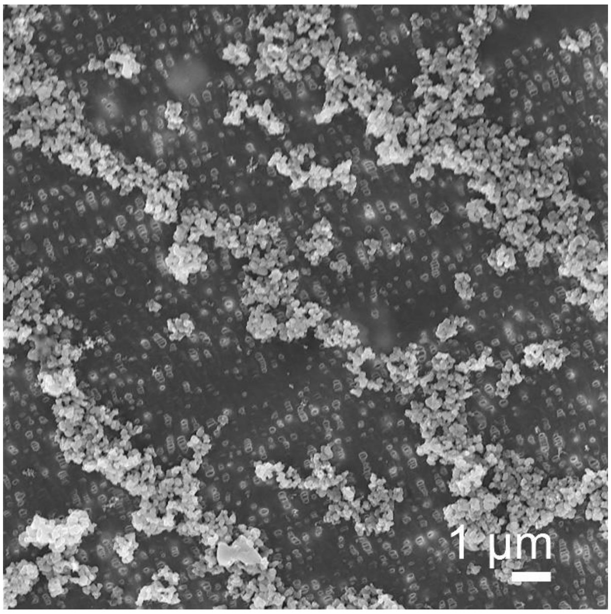

Supplementary Figure 12. SEM image of anatase TiO_2/PP separators with a surface area mass loading of 0.016 mg cm^{-2}.

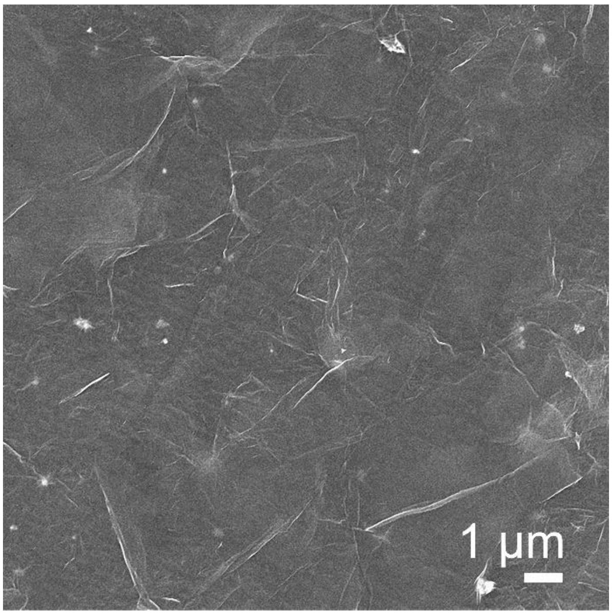

Supplementary Figure 13. SEM image of GO/PP separators with a surface area mass loading of 0.016 mg cm^{-2}.

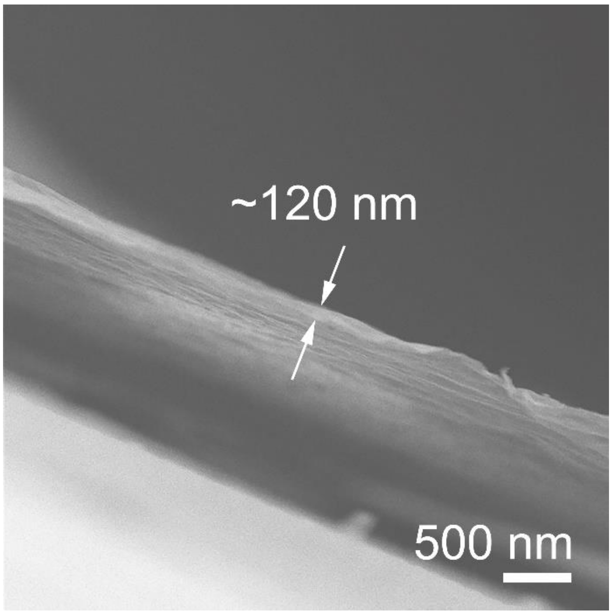

Supplementary Figure 14. Cross-section SEM image of GO/PP separators with a surface area mass loading of 0.016 mg cm^{-2}.

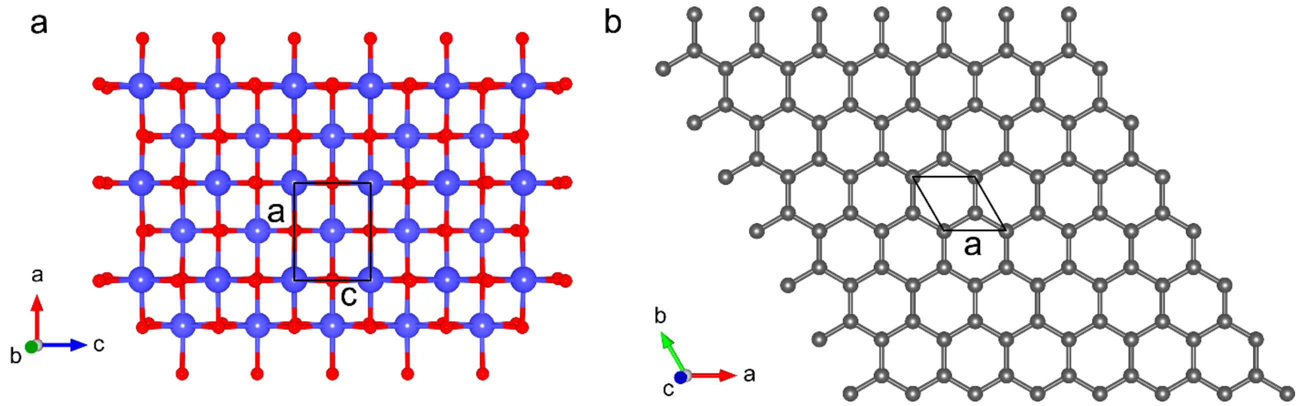

Supplementary Figure 15. 2D theoretical specific surface area of $Ti_{0.87}O_2$ and GO monolayers. (a) In-plane structure of $Ti_{0.87}O_2$ with a rectangular unit cell: a = 0.38 nm and c = 0.30 nm. (b) In-plane structure of graphene with a hexagonal unit cell: a = 0.25 nm. The ideal graphene structure was used to estimate the 2D theoretical specific surface area of GO. For an approximate calculation, the single layers of $Ti_{0.87}O_2$ and graphene were assumed to neatly deposit on the PP separator without gap. The 2D theoretical specific surface area of $Ti_{0.87}O_2$ single layer can be calculated based on the in-plane unit cell area, $W_{(Ti0.87O2)} = 2\ M_{(Ti0.87O2)} / (a \times c \times N_A)$. The 2D theoretical specific surface area of GO single layer can be calculated based on the in-plane unit cell area, $W_{(GO)} = 2\ M_{(C)} / (a \times a \times \sin 120° \times N_A)$. N_A is the Avogadro's number, $M_{(Ti0.87O2)}$ and $M_{(C)}$ are the formula weights of $Ti_{0.87}O_2$ and carbon. Under a same specific surface area, $W_{(GO)} \times n_{(GO)} = W_{(Ti0.87O2)} \times n_{(Ti0.87O2)}$. $n_{(GO)}$ and $n_{(Ti0.87O2)}$ are the number of single layers of GO and $Ti_{0.87}O_2$, respectively. So, the $n_{(GO)} / n_{(Ti0.87O2)}$ = ~2.9. Considering the crystallinity thickness of GO and $Ti_{0.87}O_2$ is 0.34 and 0.75 nm, respectively. The thickness (h) of the functional layer of GO and $Ti_{0.87}O_2$ with the same specific surface area is $h_{(GO)} / h_{(Ti0.87O2)}$ = ~1.36.

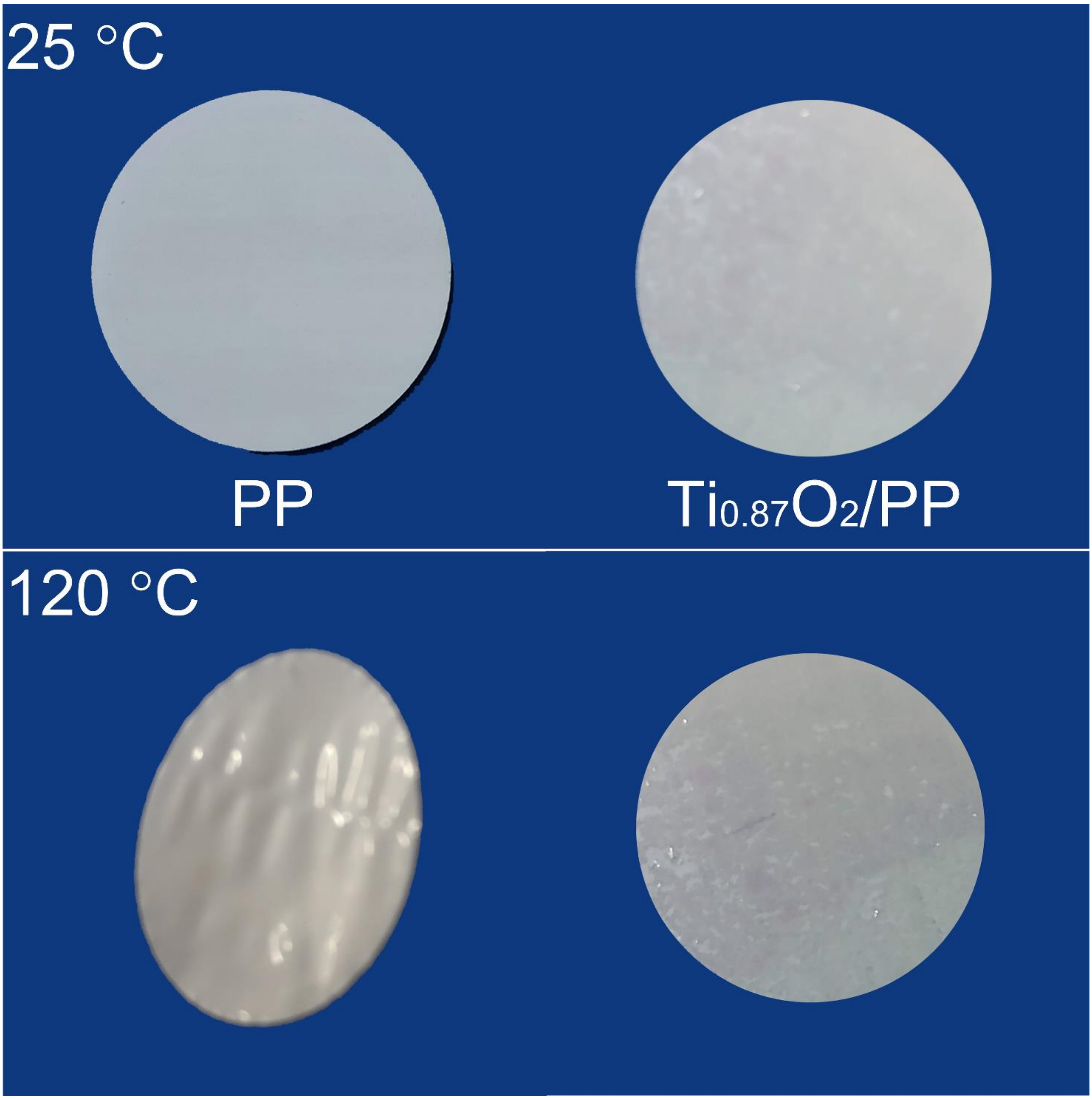

Supplementary Figure 16. Photographic pictures of the PP and $Ti_{0.87}O_2$/PP separators before and after heating process.

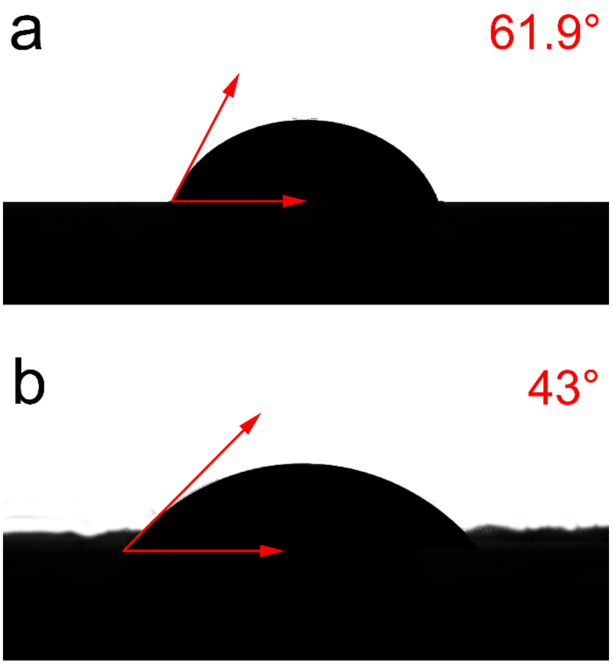

Supplementary Figure 17. Contact angle measurements for electrolytes (1 M LiTFSI in DME: DOL 1: 1, v/v) on (a) PP and (b) $Ti_{0.87}O_2$/PP separators.

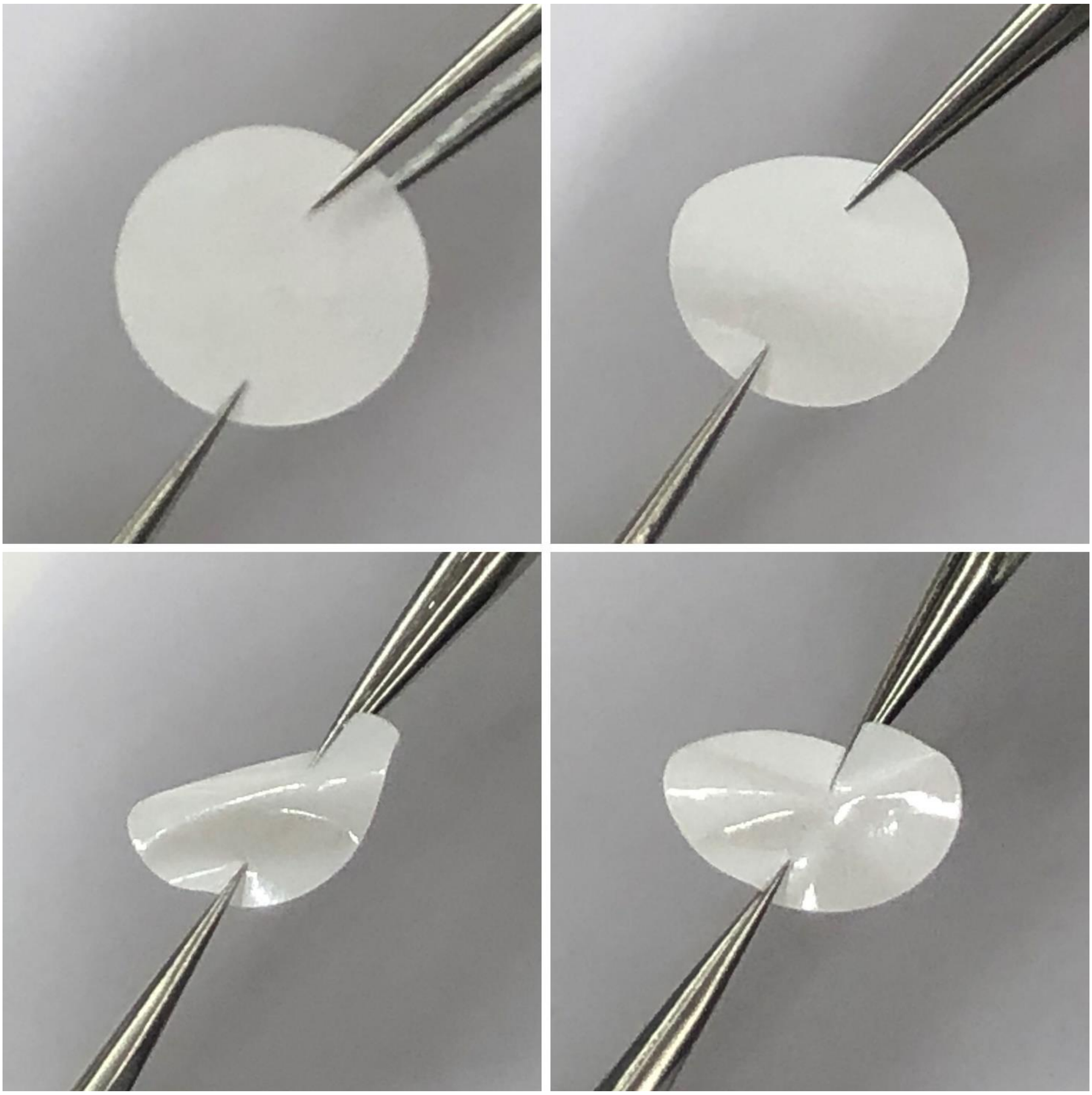

Supplementary Figure 18. Digital photos of the $Ti_{0.87}O_2$/PP separator under different bending conditions.

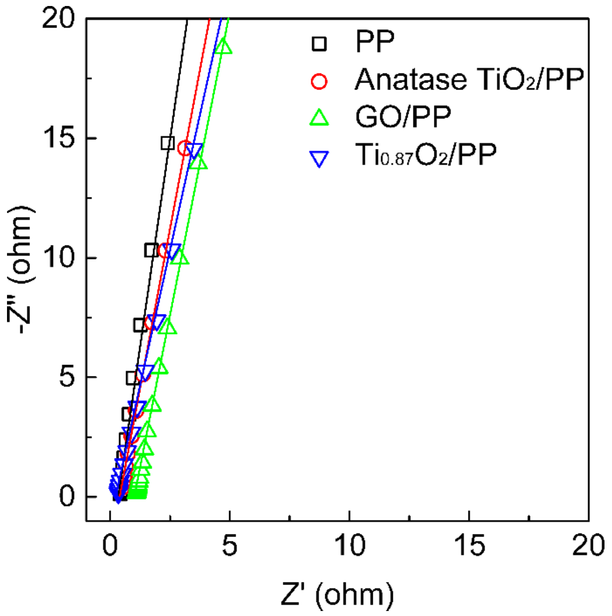

Supplementary Figure 19. Nyquist plots of PP, anatase TiO_2/PP, GO/PP and $Ti_{0.87}O_2$/PP separators estimating the Li-ion conductivity.

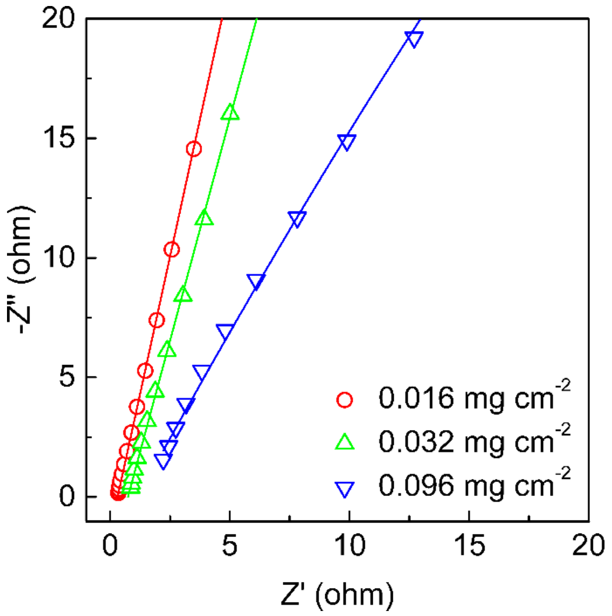

Supplementary Figure 20. Nyquist plots of $Ti_{0.87}O_2$/PP separators with different weight densities.

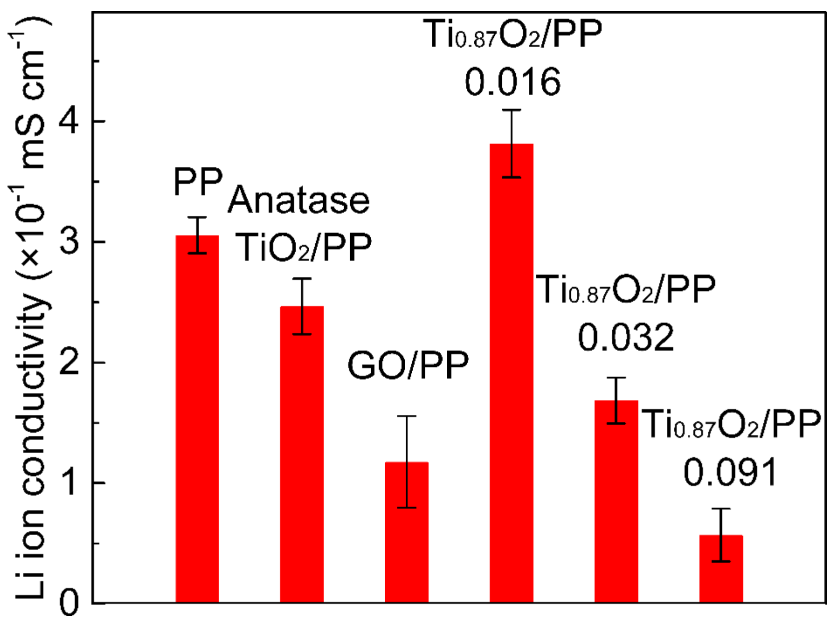

Supplementary Figure 21. Li ion conductivity of PP, anatase TiO_2/PP, GO/PP and $Ti_{0.87}O_2$/PP separators with different surface area mass loadings. Error bars were included, which represent the standard deviation of the data taken from five samples.

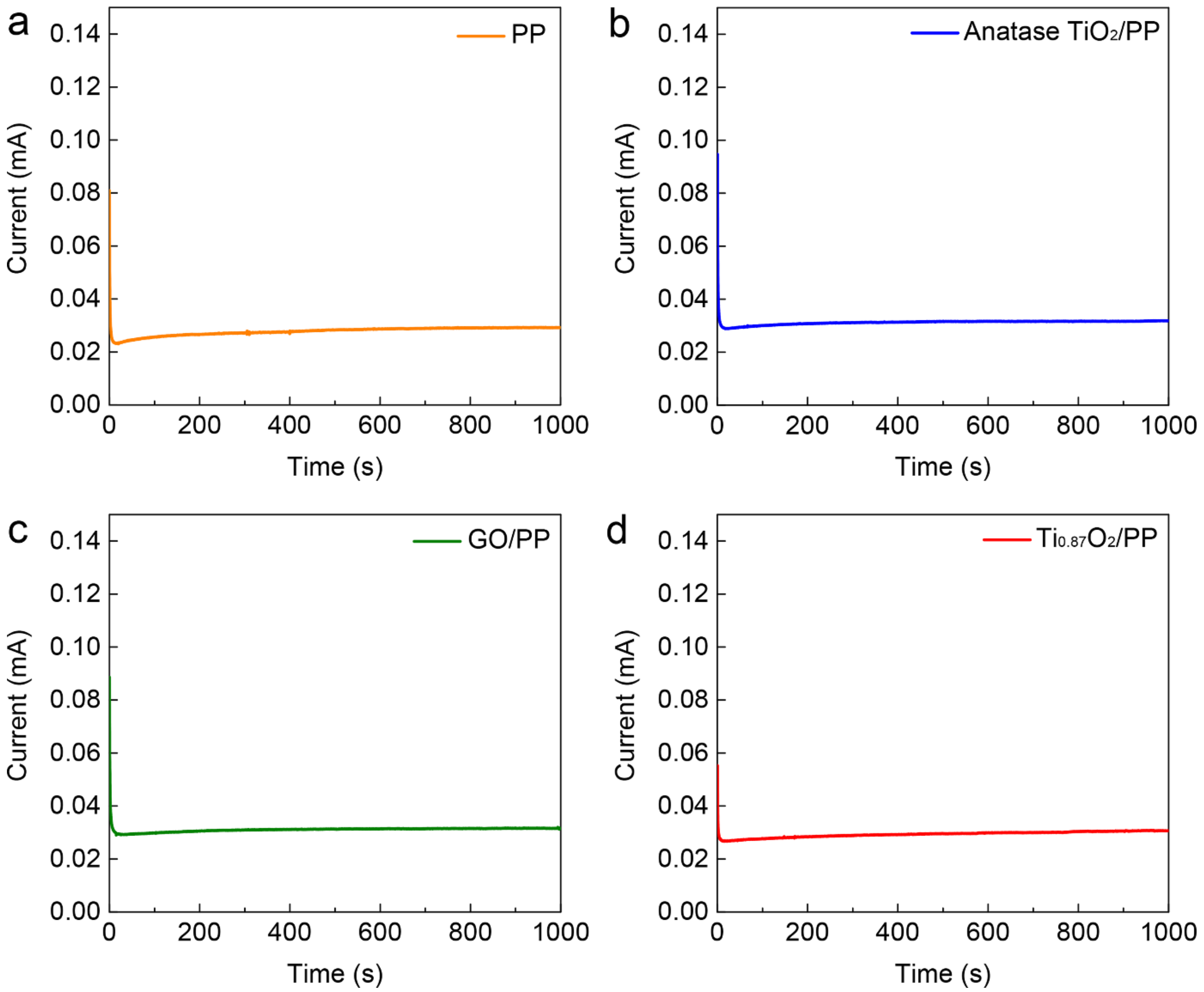

Supplementary Figure 22. Chronoamperometric measurements of PP, anatase TiO_2/PP, GO/PP and $Ti_{0.87}O_2$/PP separators.

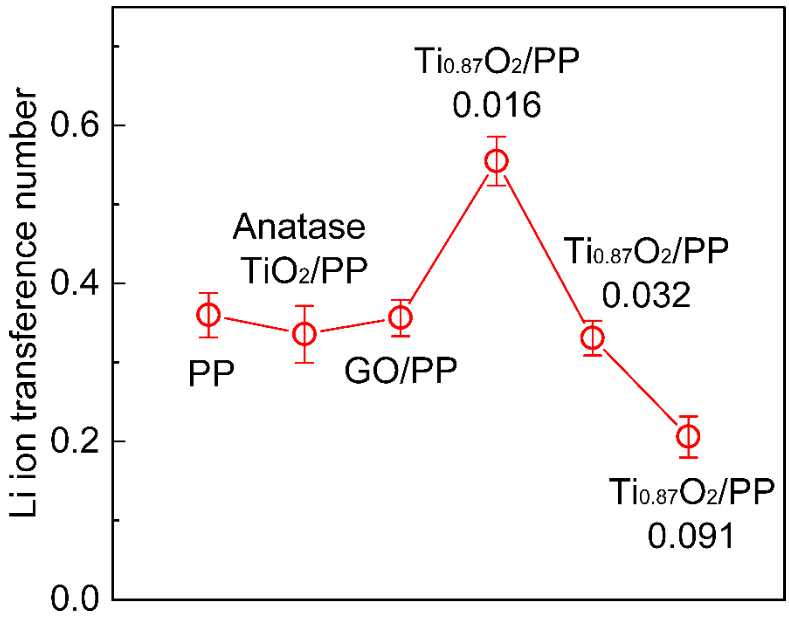

Supplementary Figure 23. Li ion transference number of PP, anatase TiO_2/PP, GO/PP and $Ti_{0.87}O_2$/PP separators with different surface area mass loadings. Error bars were included, which represent the standard deviation of the data taken from five samples.

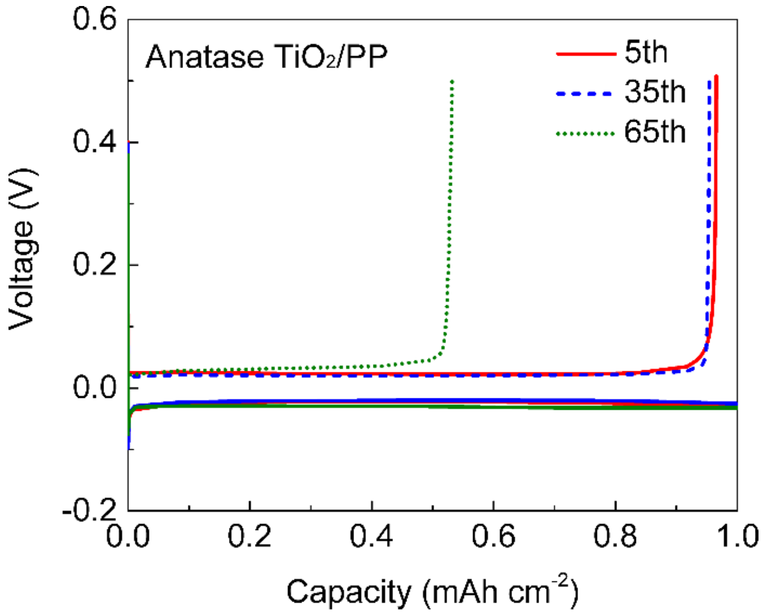

Supplementary Figure 24. Voltage profiles of Li plating/stripping processes in Li||Cu cells with anatase TiO_2/PP separators with an areal capacity of 1 mAh cm^{-2} at 1 mA cm^{-2}.

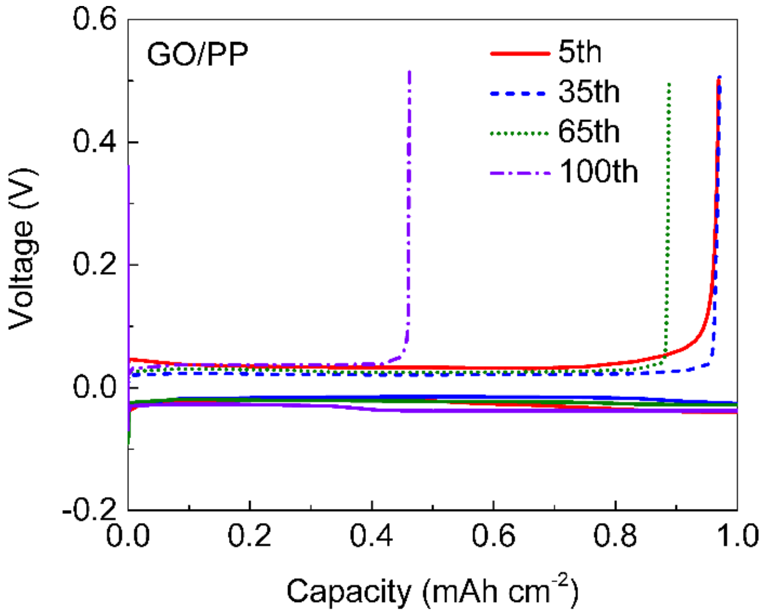

Supplementary Figure 25. Voltage profiles of Li plating/stripping processes in Li||Cu cells with GO/PP separators with an areal capacity of 1 mAh cm^{-2} at 1 mA cm^{-2}.

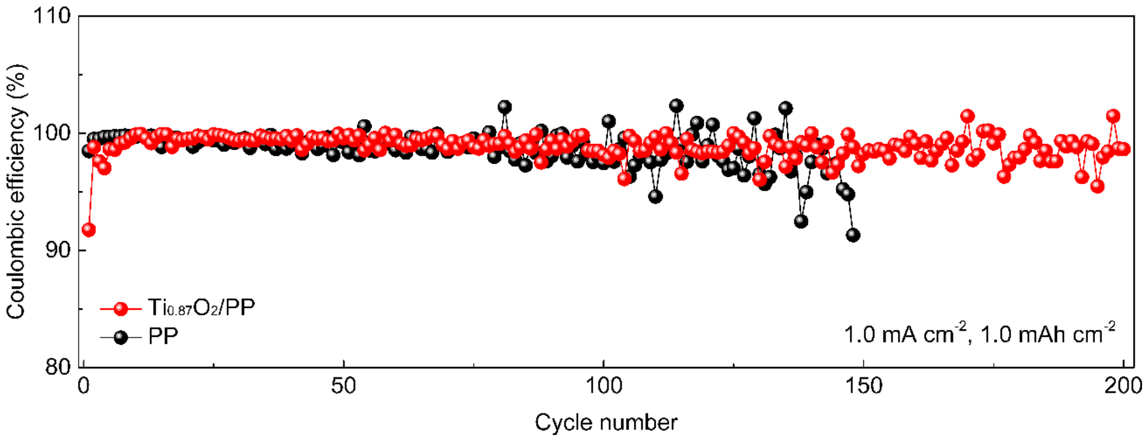

Supplementary Figure 26. Coulombic efficiencies of Na||Cu cells with PP and $Ti_{0.87}O_2$/PP separators with an area capacity of 1 mAh cm^{-2} at 1 mA cm^{-2}.

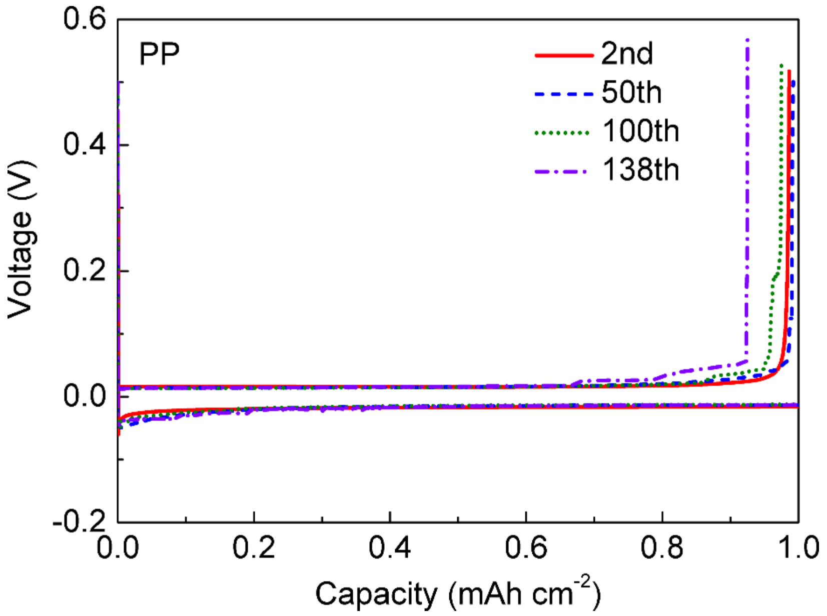

Supplementary Figure 27. Voltage profiles of Na plating/stripping processes in Na||Cu cells with PP separators with an areal capacity of 1 mAh cm^{-2} at 1 mA cm^{-2}.

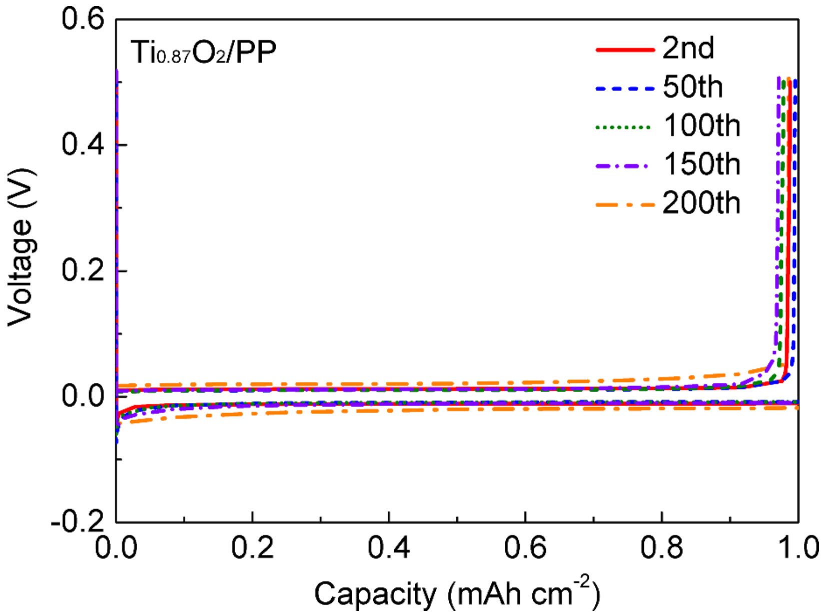

Supplementary Figure 28. Voltage profiles of Na plating/stripping processes in Na||Cu cells with $Ti_{0.87}O_2$/PP separators with an areal capacity of 1 mAh cm^{-2} at 1 mA cm^{-2}.

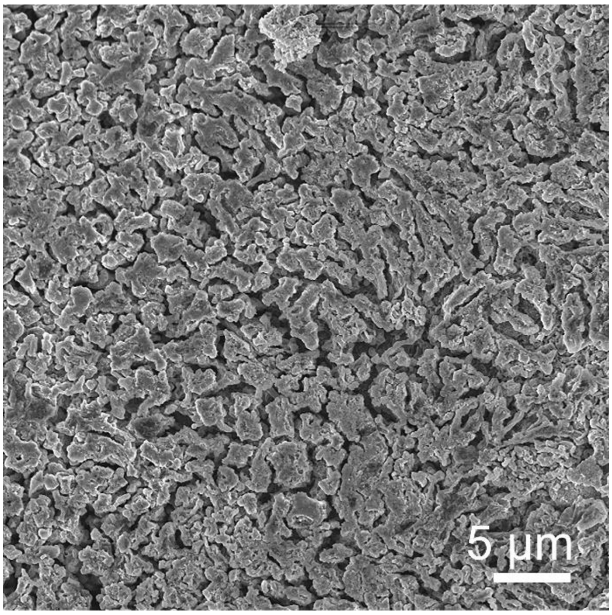

Supplementary Figure 29. SEM image of the Li metal anodes disassembled from the symmetrical cell with the PP separator at a current density of 2 mA cm^{-2} with a capacity of 1 mAh cm^{-2} for 20 cycles.

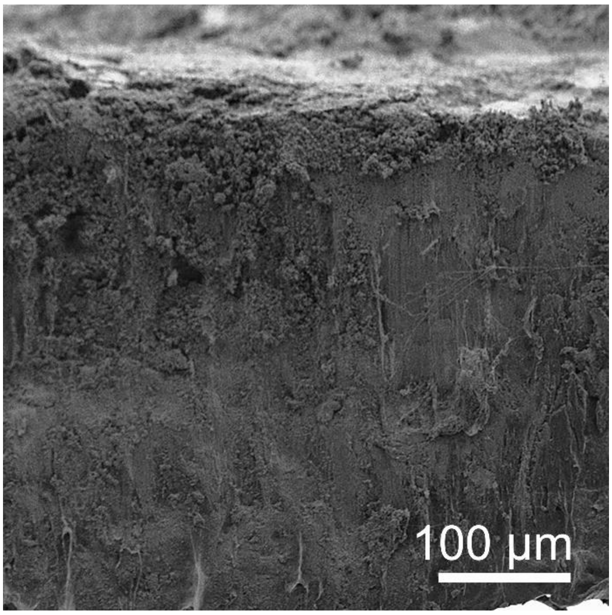

Supplementary Figure 30. Cross-section SEM image of the Li metal anodes disassembled from the symmetrical cell with the PP separator at a current density of 2 mA cm^{-2} with a capacity of 1 mAh cm^{-2} for 20 cycles.

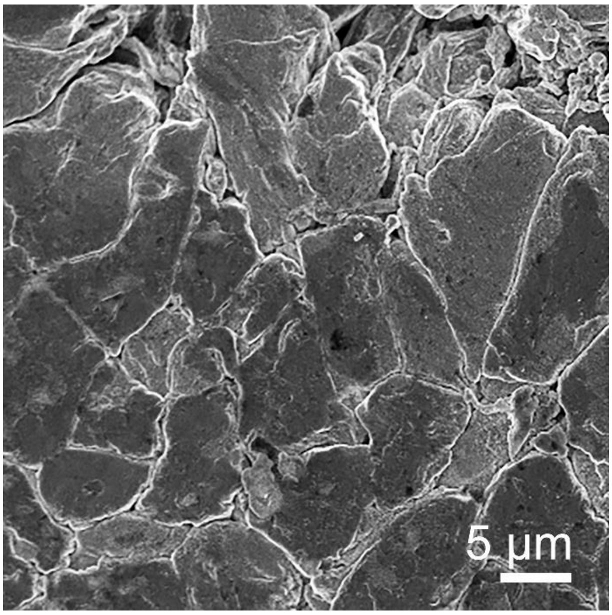

Supplementary Figure 31. SEM image of the Li metal anodes disassembled from the symmetrical cell with the $Ti_{0.87}O_2$/PP separator at a current density of 2 mA cm^{-2} with a capacity of 1 mAh cm^{-2} for 20 cycles.

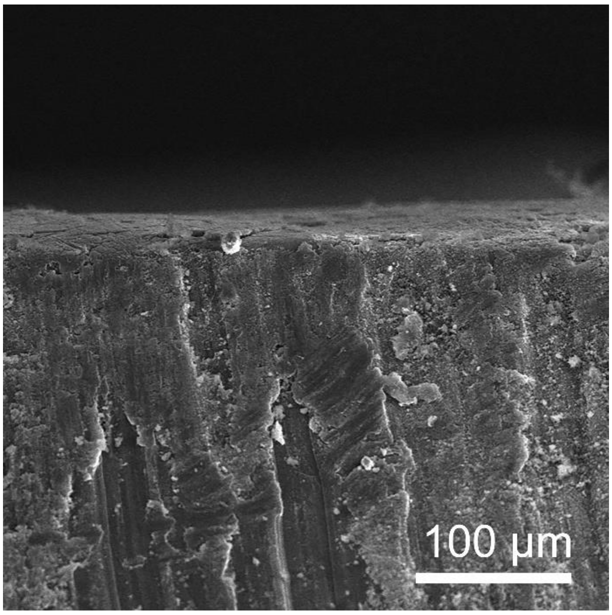

Supplementary Figure 32. Cross-section SEM image of the Li metal anodes disassembled from the symmetrical cell with the $Ti_{0.87}O_2$/PP separator at a current density of 2 mA cm^{-2} with a capacity of 1 mAh cm^{-2} for 20 cycles.

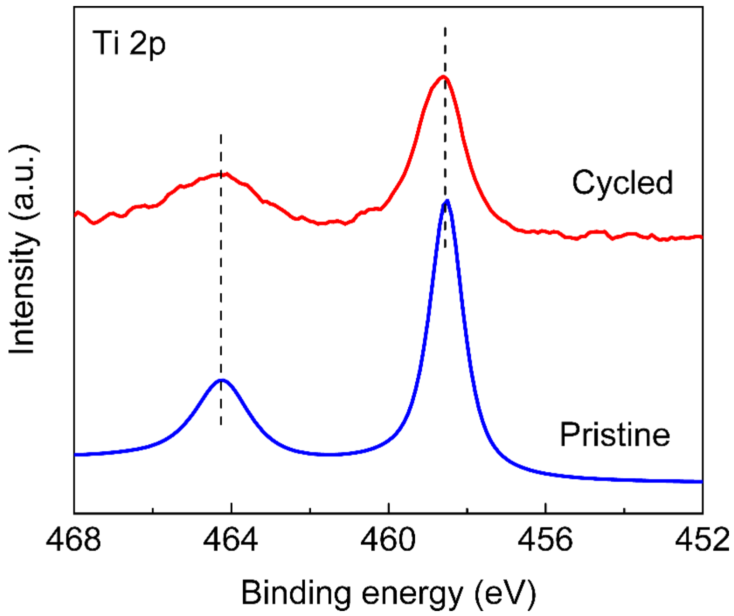

Supplementary Figure 33. High-resolution XPS spectrum of Ti 2p of pristine and cycled $Ti_{0.87}O_2$/PP separators disassembled from the symmetrical cell at a current density of 2 mA cm^{-2} with a capacity of 1 mAh cm^{-2} for 20 cycles.

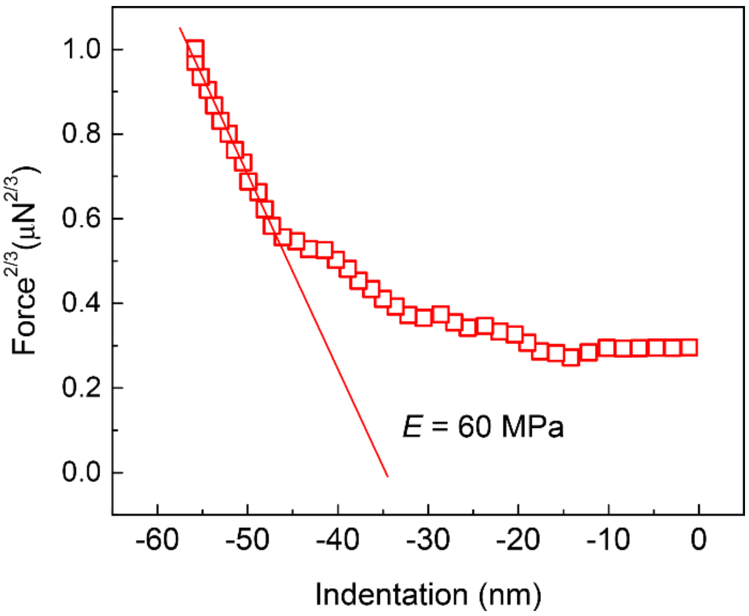

Supplementary Figure 34. A representative force-indentation curve of the $Ti_{0.87}O_2$/PP separator. The curve is fitted using the Hertzian model in the linear region.

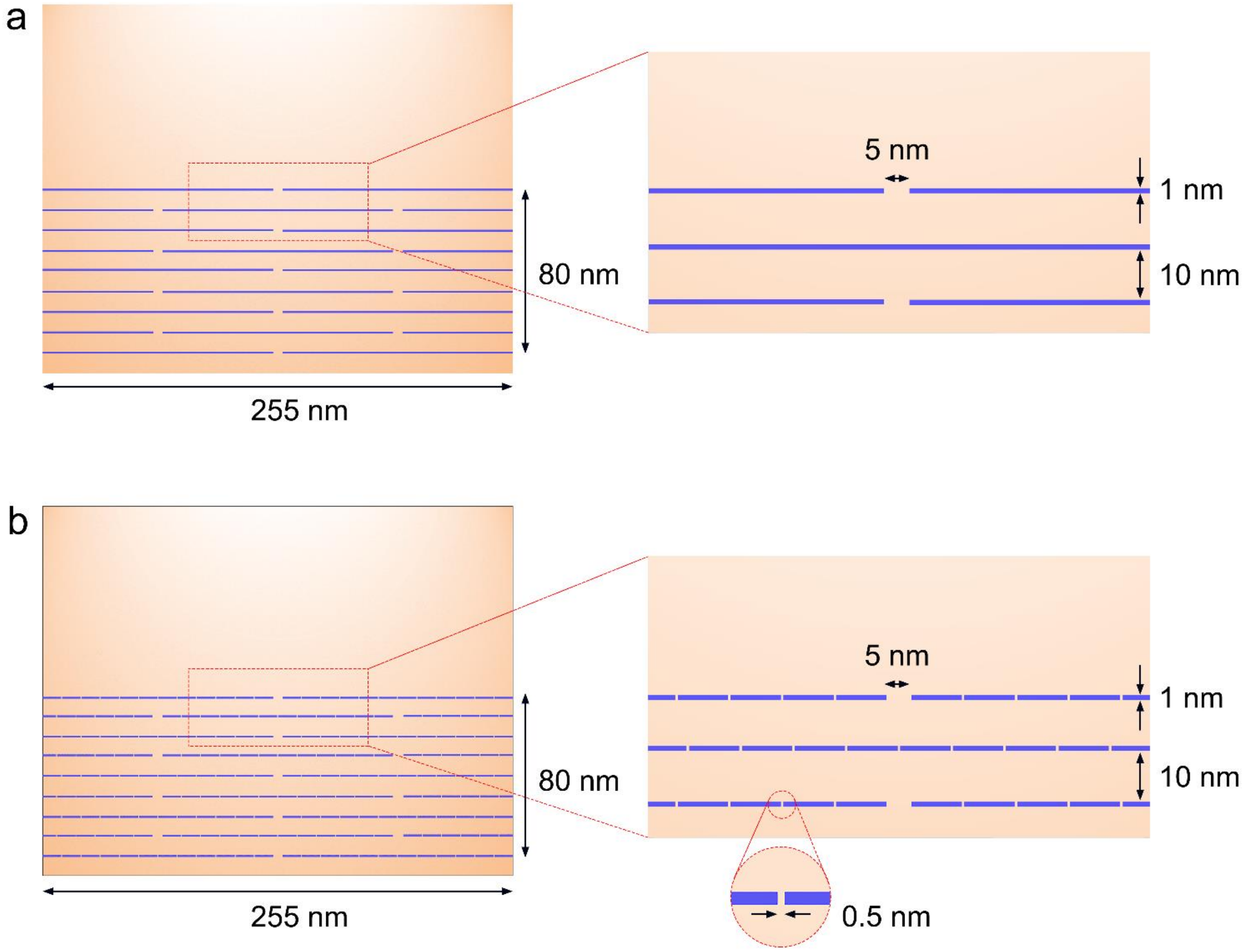

Supplementary Figure 35. The models of restacked thin layers for the (a) conventional nanosheets (without defects) and (b) defective nanosheets.

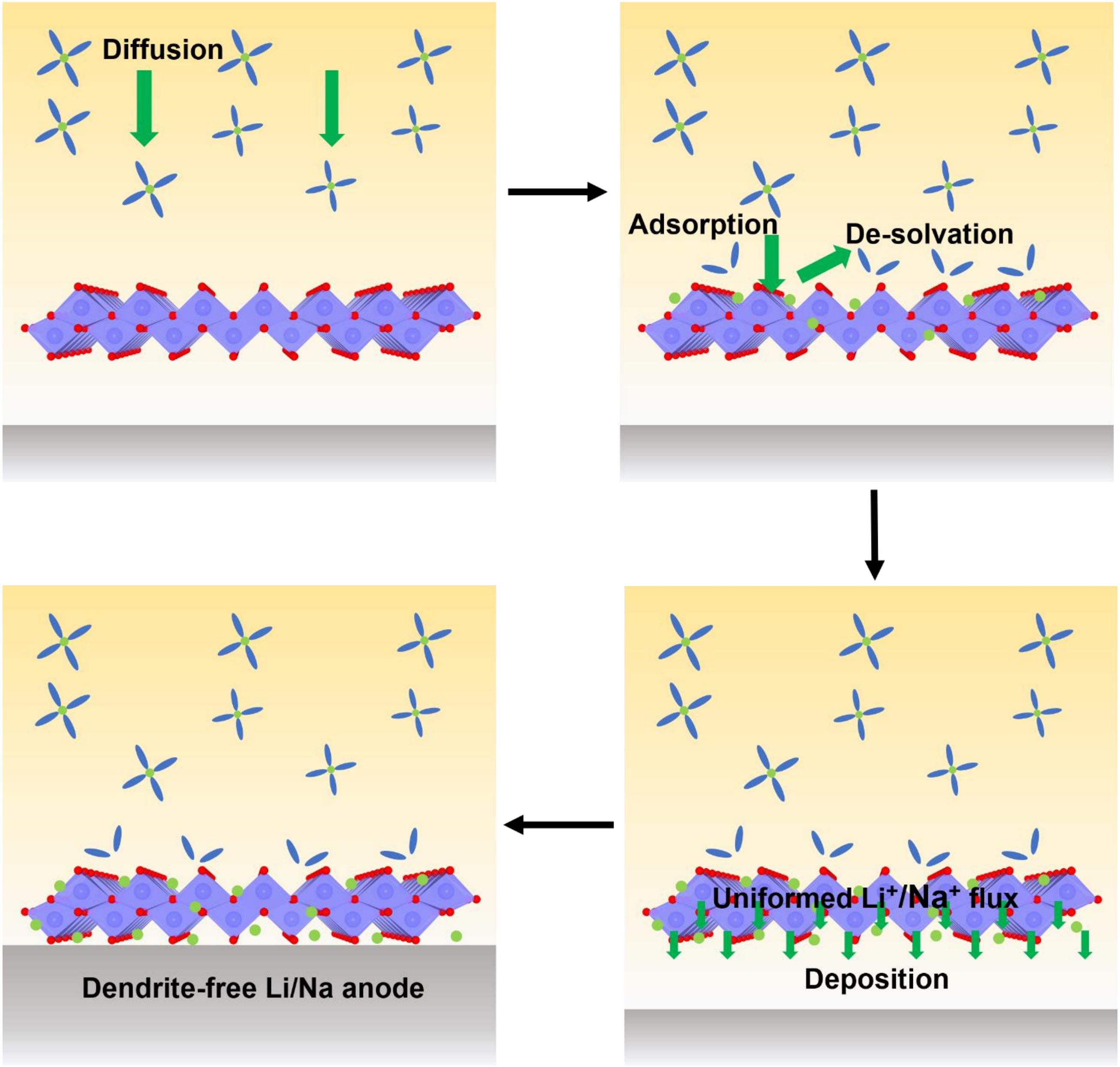

Supplementary Figure 36. Schematic illustration of mechanism of dendrite-free Li/Na anode by using anionic $Ti_{0.87}O_2$ nanosheets with atomic Ti vacancies.

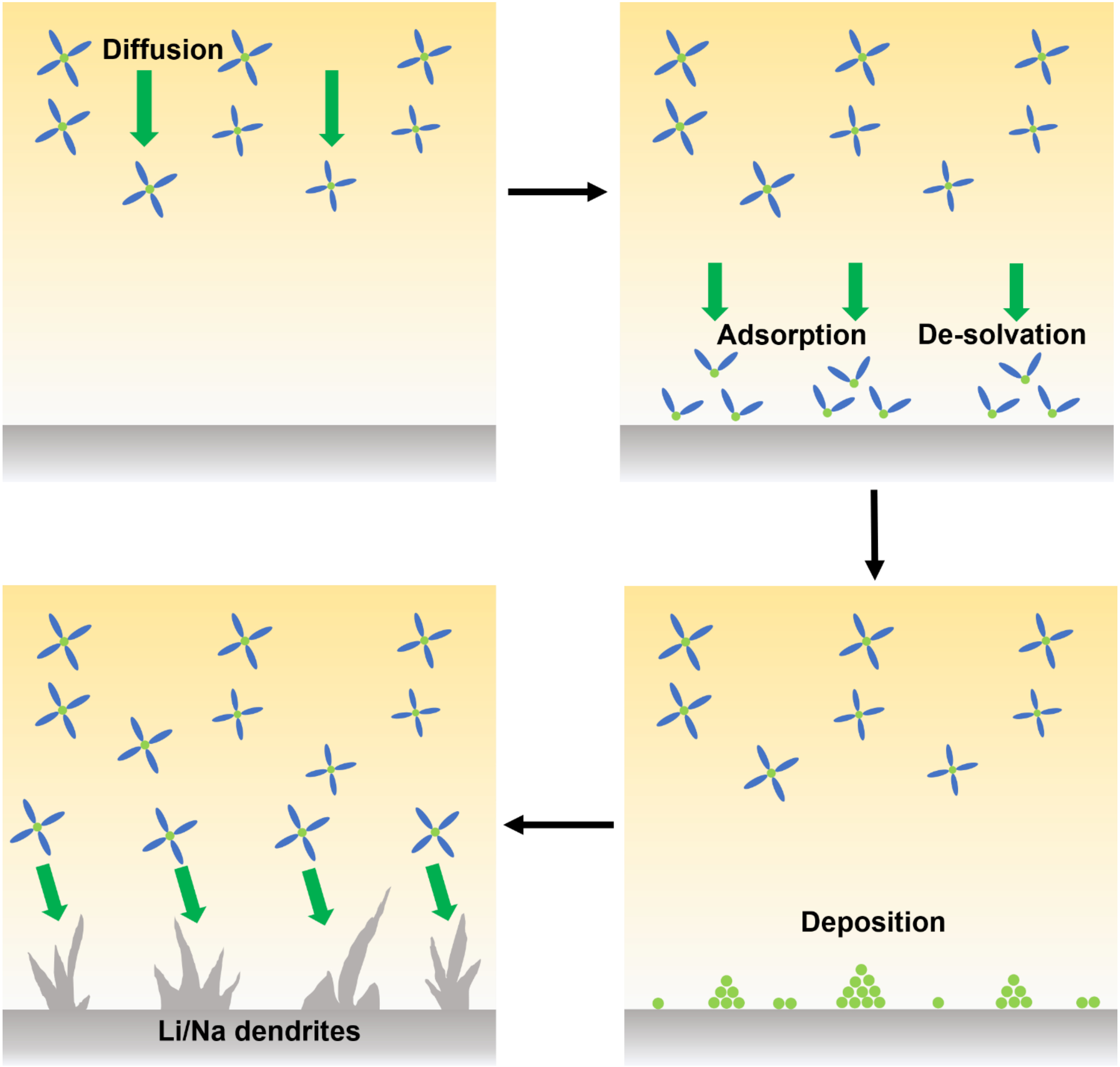

Supplementary Figure 37. Schematic illustration of Li/Na deposition over the bare anode.

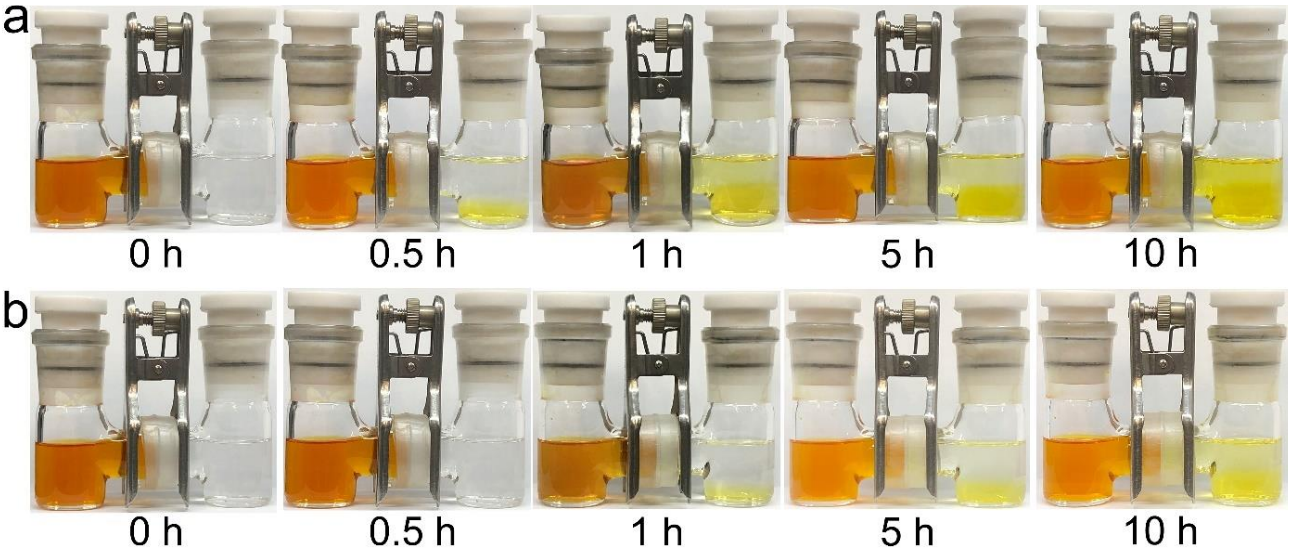

Supplementary Figure 38. Polysulfide permeation measurements in H-type cells with the (a) anatase TiO_2/PP and (b) GO/PP separators.

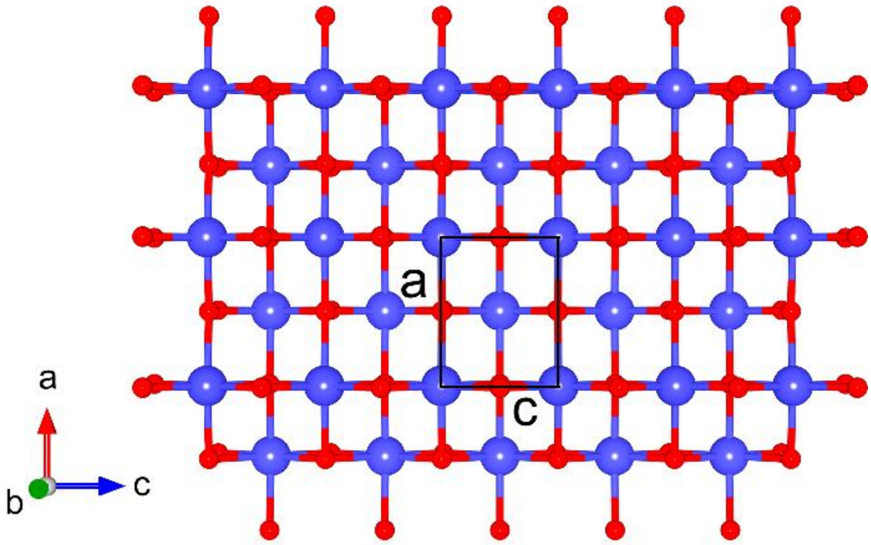

Supplementary Figure 39. Calculation of negative charge density of $Ti_{0.87}O_2^{0.52-}$ nanosheets. In-plane structure of $Ti_{0.87}O_2$ shows a rectangular unit cell with a = 0.38 nm and c = 0.30 nm. The 2D charge density (ρ) of $Ti_{0.87}O_2$ can be calculated based on the in-plane unit cell area, $\rho_{(Ti0.87O2)} = 2 \times 0.52 \times 1.60 \times 10^{-19} / (a \times c) = 1.46$ C m^{-2}.

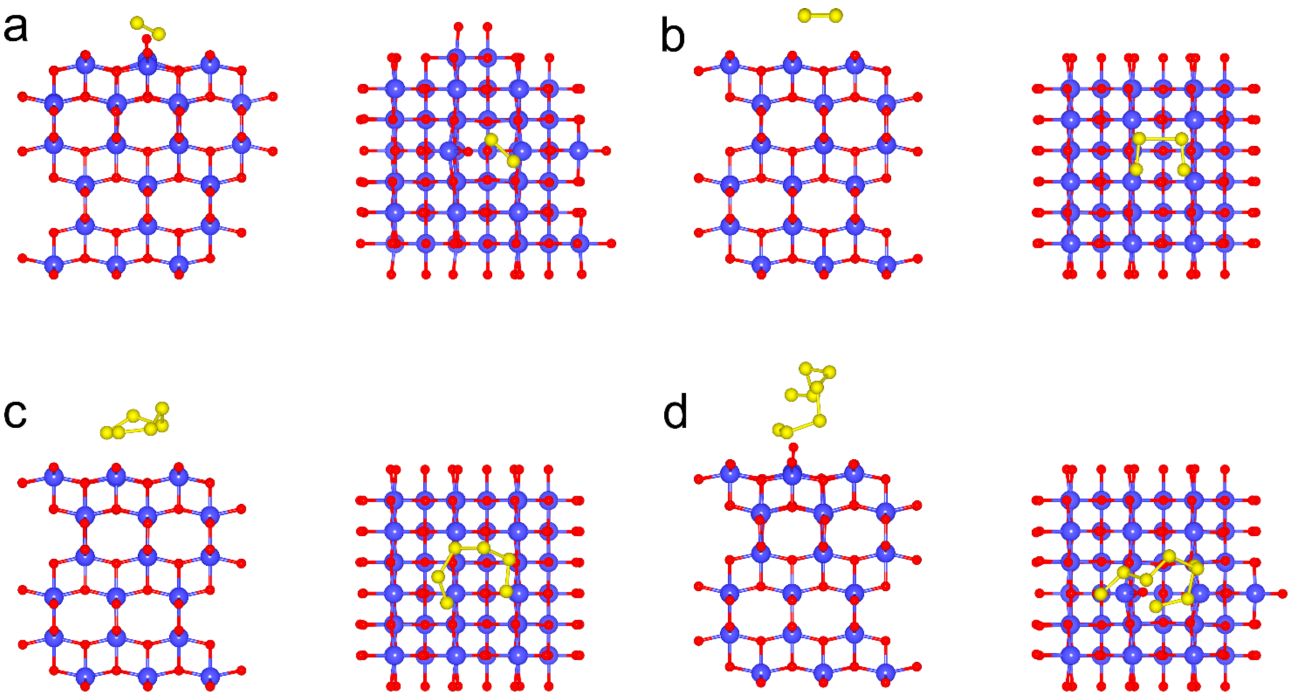

Supplementary Figure 40. Optimized conformations of (a) S_2^{2-}, (b) S_4^{2-}, (c) S_6^{2-} and (d) S_8^{2-} on anatase TiO_2.

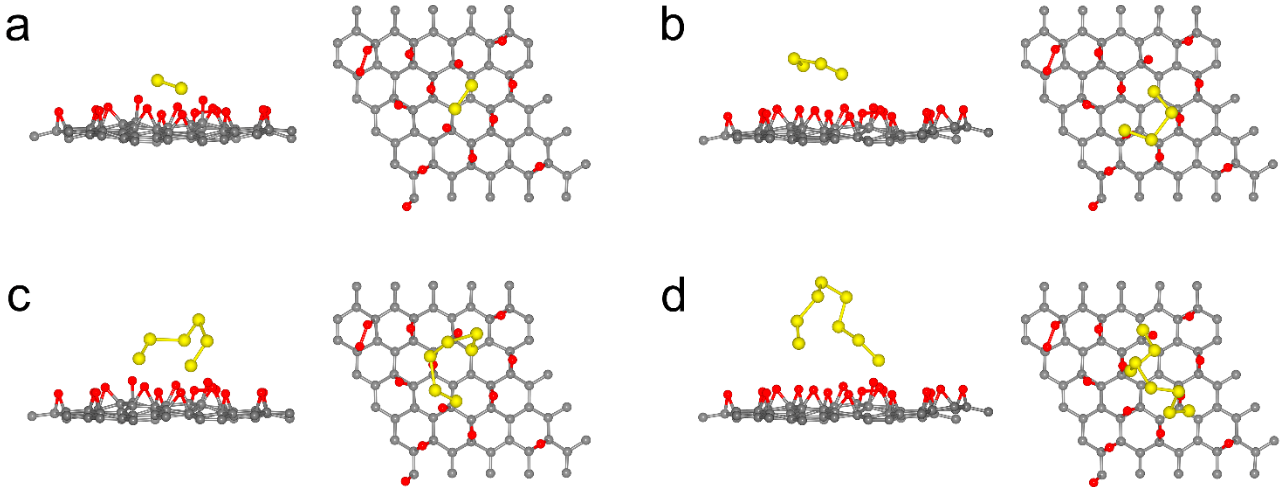

Supplementary Figure 41. Optimized conformations of (a) S_2^{2-}, (b) S_4^{2-}, (c) S_6^{2-} and (d) S_8^{2-} on GO sheet.

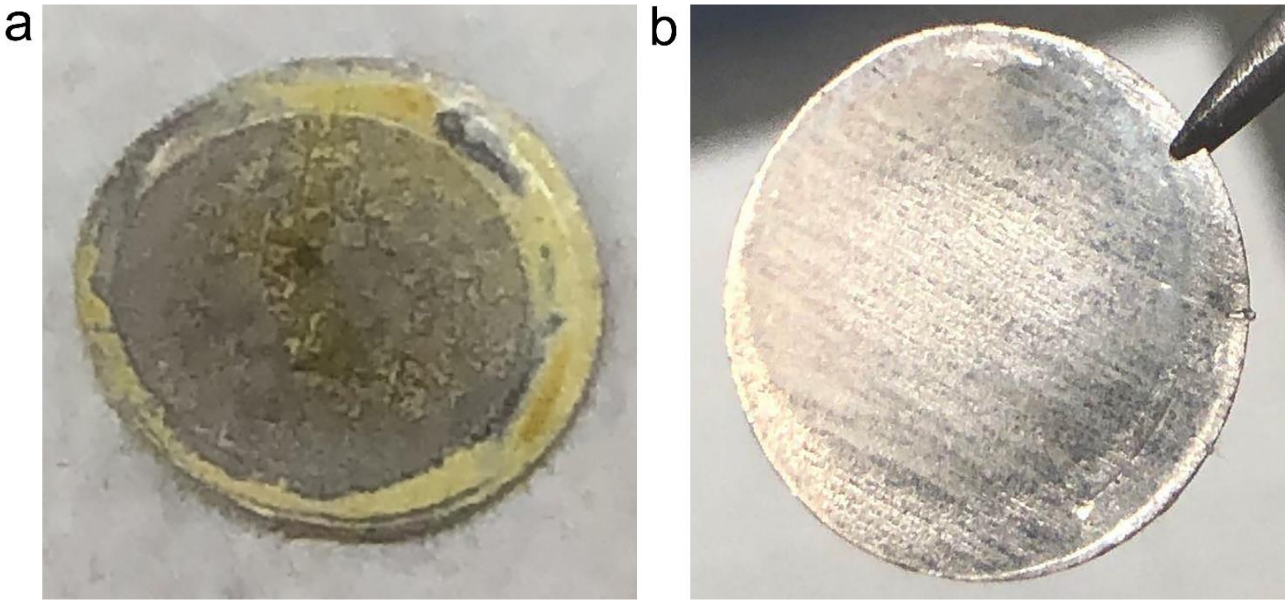

Supplementary Figure 42. Digital images of the Li metal anodes of the disassembled cells after10 cycles with the (a) PP and (b) $Ti_{0.87}O_2$/PP separators.

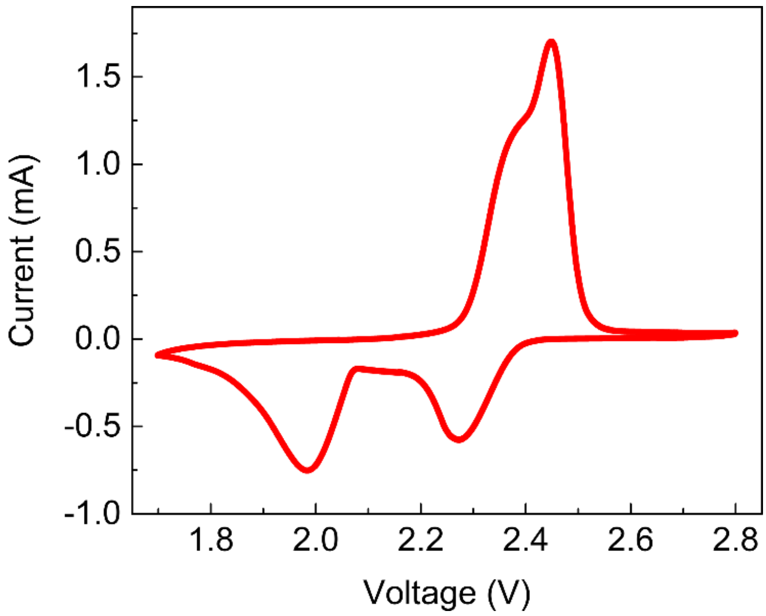

Supplementary Figure 43. CV curve of the Li–S cell with a $Ti_{0.87}O_2$/PP separator at 0.1 mV s^{-1}.

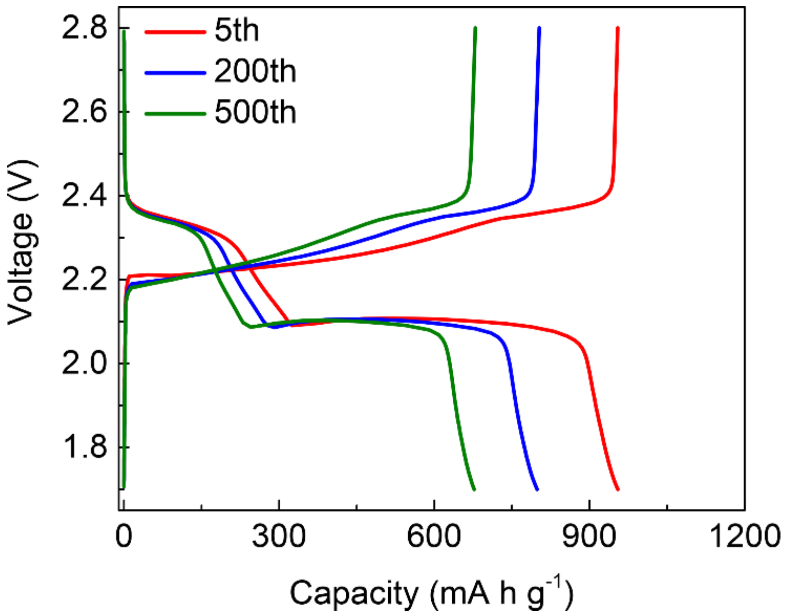

Supplementary Figure 44. Voltage profiles of the Li–S cell with a $Ti_{0.87}O_2$/PP separator at 0.2C.

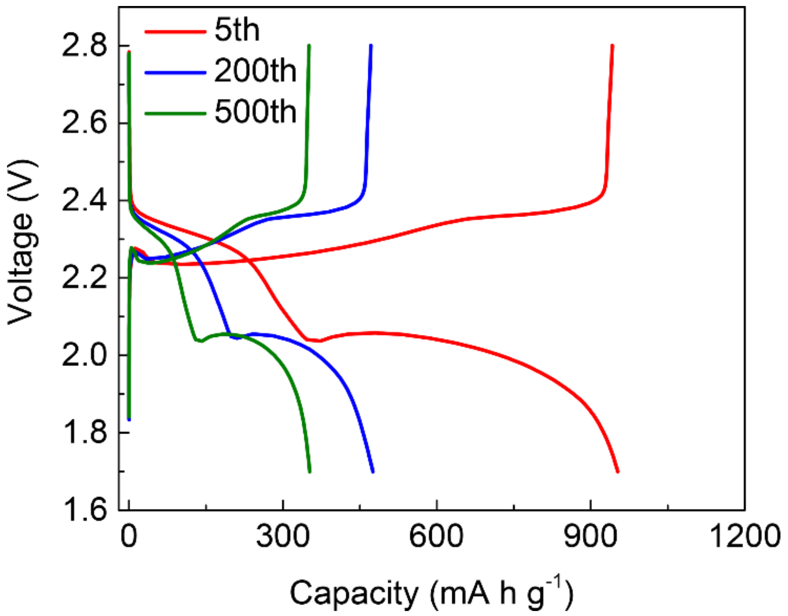

Supplementary Figure 45. Voltage profiles of the Li–S cell with a PP separator at 0.2C.

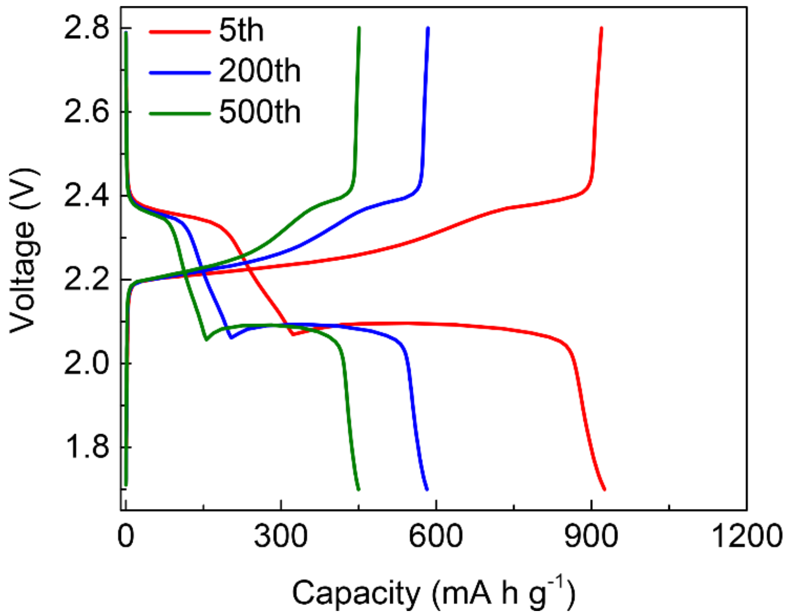

Supplementary Figure 46. Voltage profiles of the Li–S cell with an anatase TiO_2/PP separator at 0.2C.

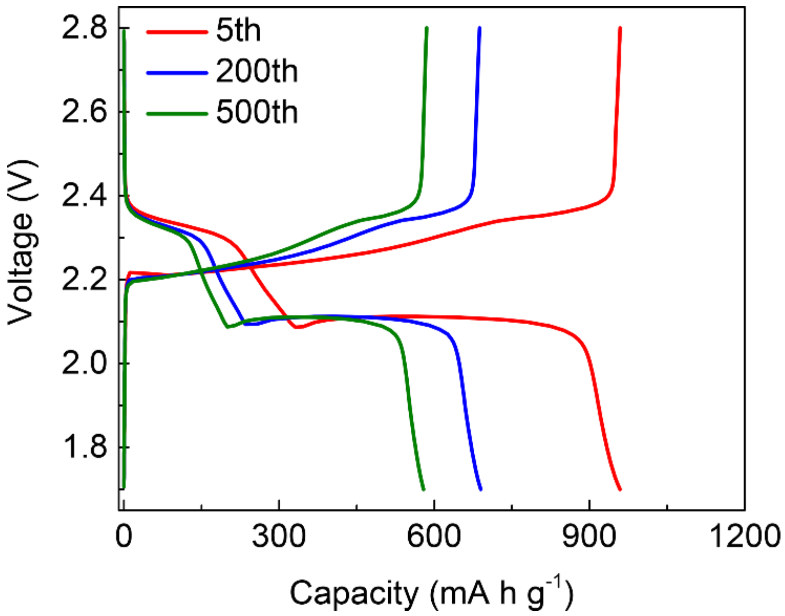

Supplementary Figure 47. Voltage profiles of the Li–S cell with a GO/PP separator at 0.2C.

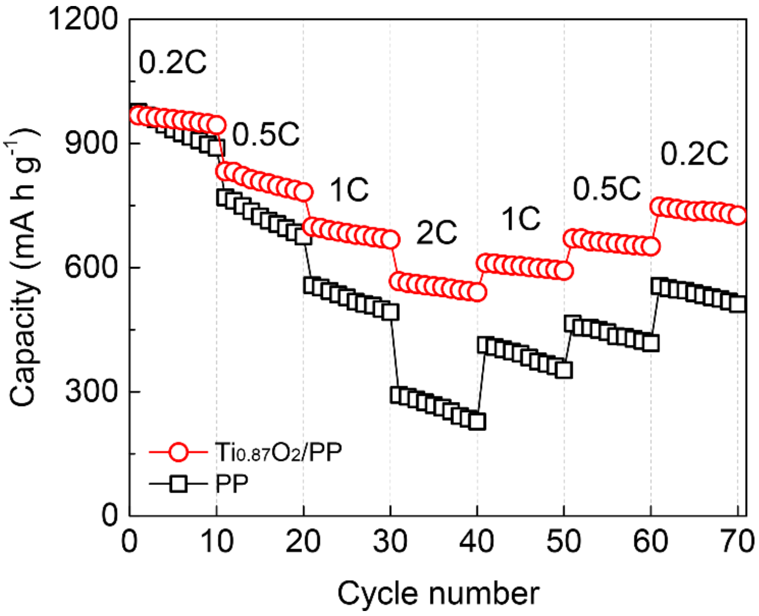

Supplementary Figure 48. The rate performance of Li–S cells with PP and $Ti_{0.87}O_2$/PP separators.

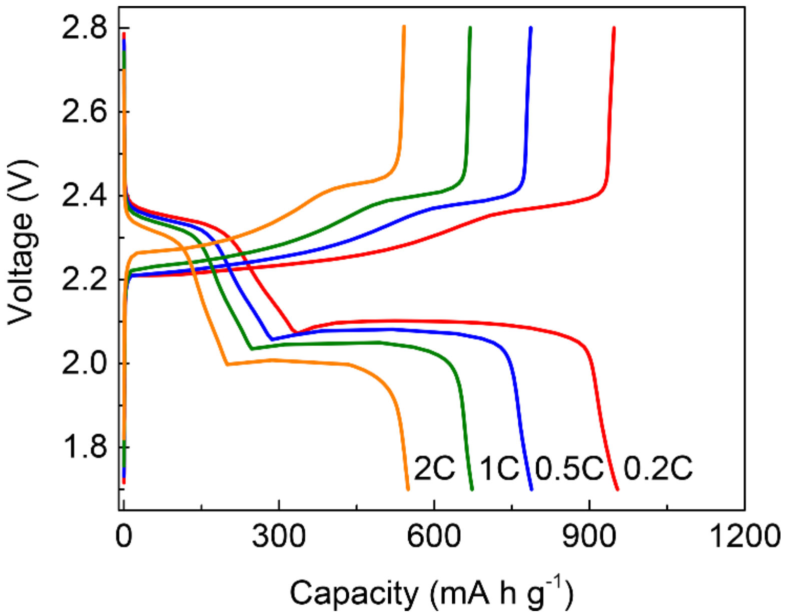

Supplementary Figure 49. Voltage profiles of the Li–S cell with a $Ti_{0.87}O_2$/PP separator at various C rates.

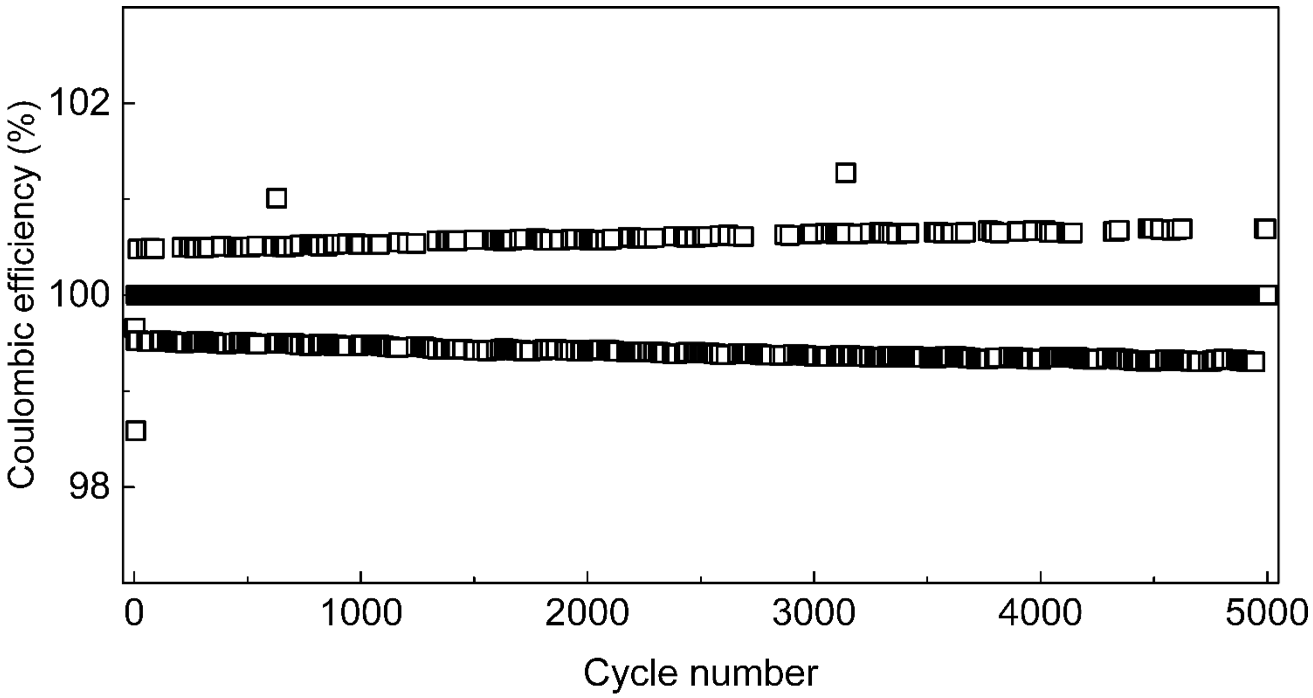

Supplementary Figure 50. Coulombic efficiency for a Li-S cell with a $Ti_{0.87}O_2$/PP separator during the long-term cycling at 1C for 5000 cycles.

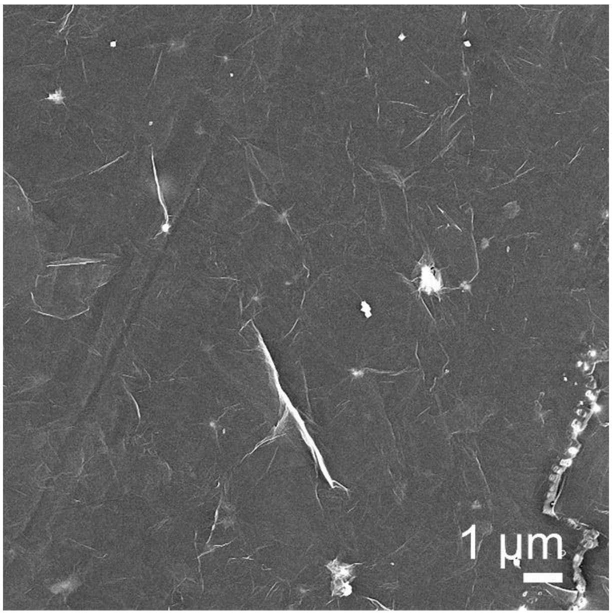

Supplementary Figure 51. SEM image of the cycled $Ti_{0.87}O_2$/PP separators from the disassembled cells in a fully discharged state after 500 cycles.

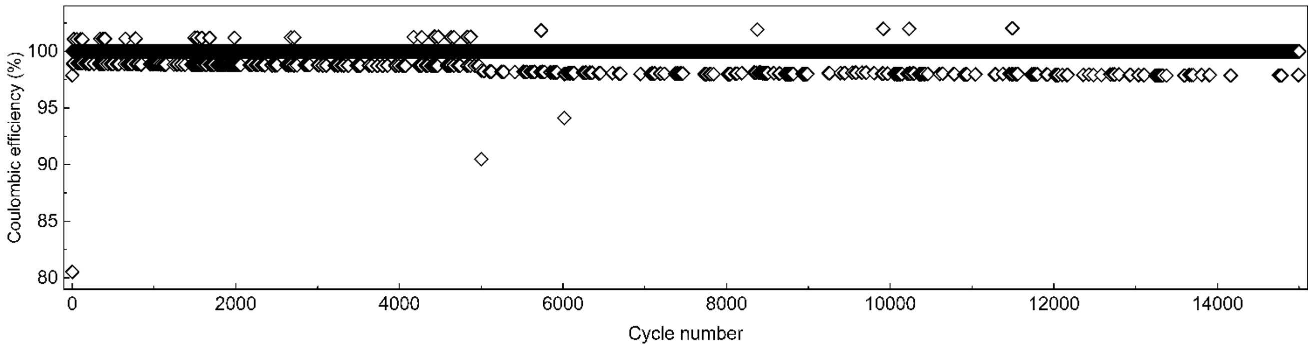

Supplementary Figure 52. Coulombic efficiency for a Li-S cell with a $Ti_{0.87}O_2$/PP separator at a sulfur mass loading of 3.5 mg cm^{-2} during the long-term cycling.

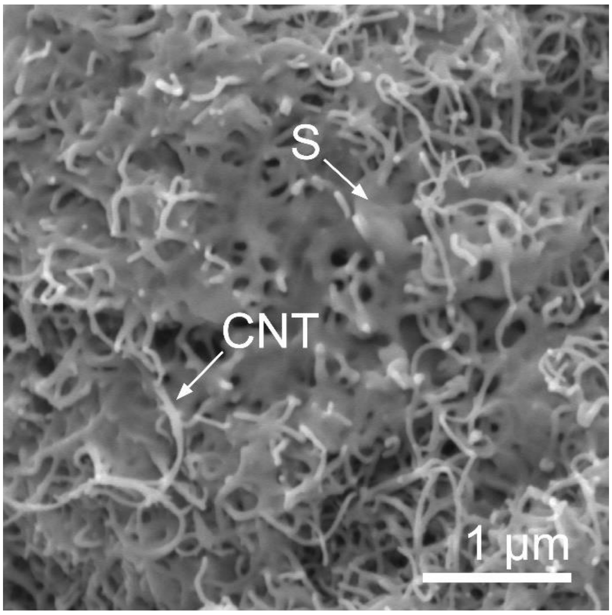

Supplementary Figure 53. SEM image of the CNT/S cathodes.

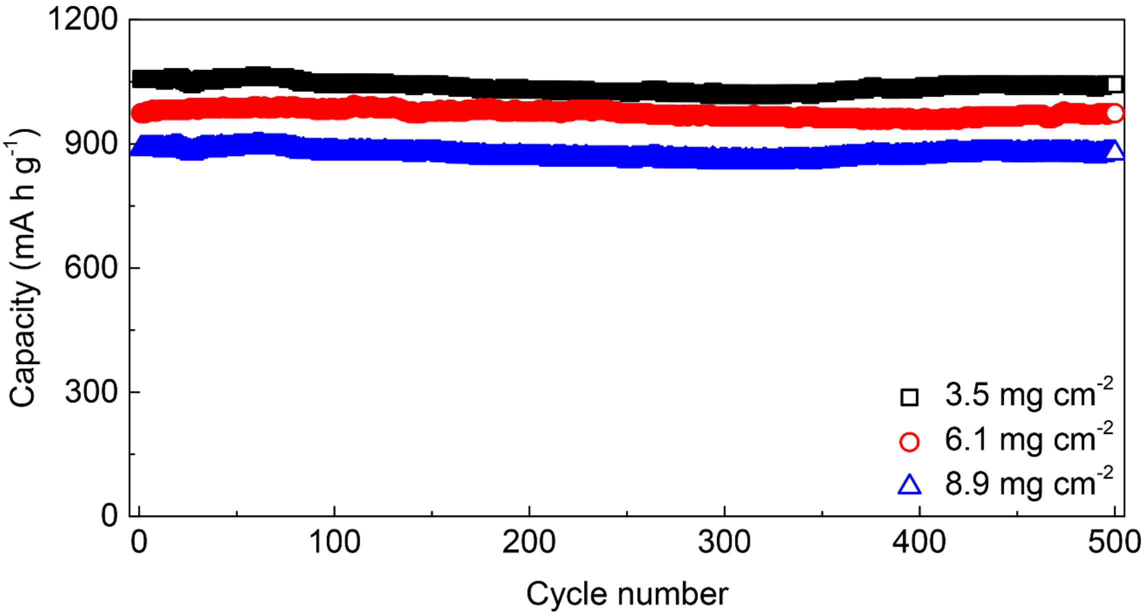

Supplementary Figure 54. Cycling performance of the Li-S cells at 0.2C using the CNT/S cathodes and the $Ti_{0.87}O_2$/PP separators.

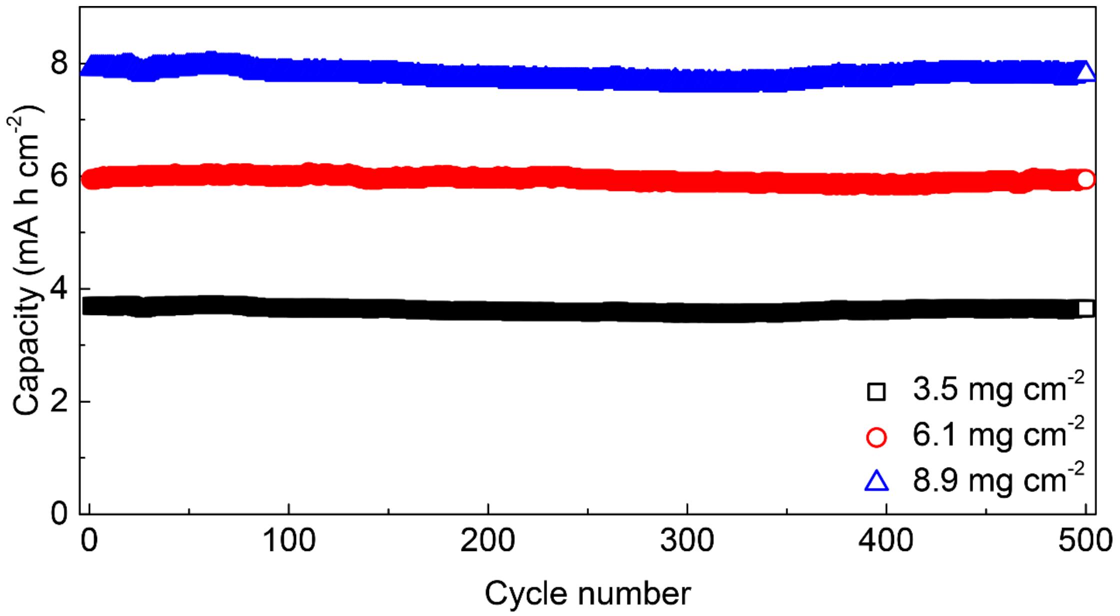

Supplementary Figure 55. Areal capacities of the Li-S cells using the CNT/S cathodes and the $Ti_{0.87}O_2$/PP separators.

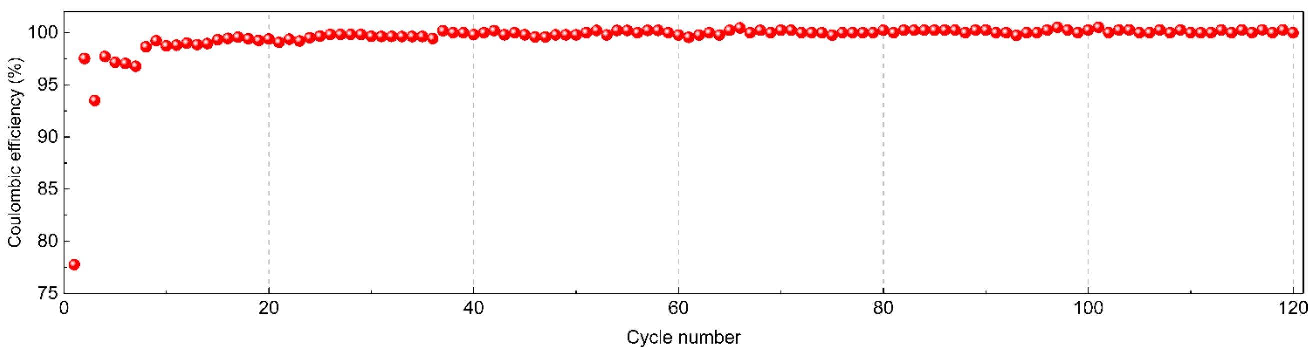

Supplementary Figure 56. Coulombic efficiency for a flexible Li-S pouch cell with a $Ti_{0.87}O_2$/PP separator under different bending angles during the cycling test.

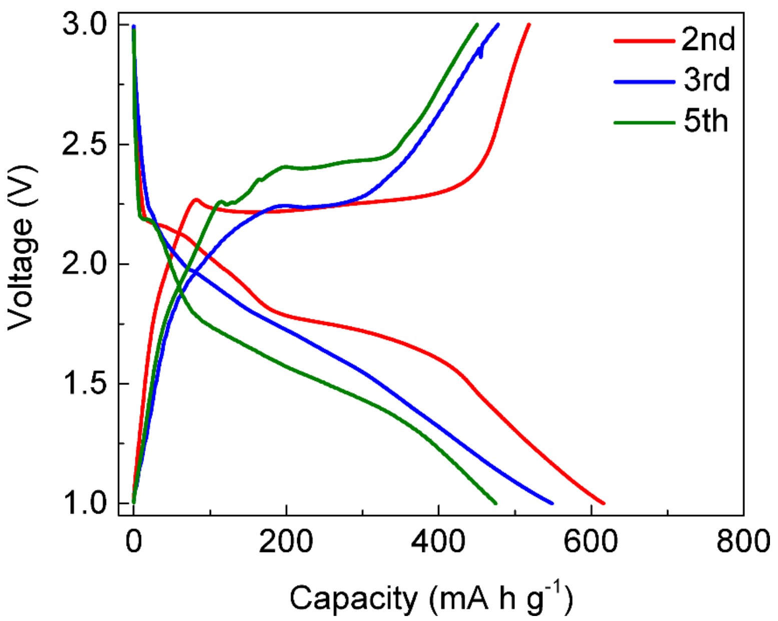

Supplementary Figure 57. Voltage profiles of the Li–Se cell with a PP separator at 0.2C.

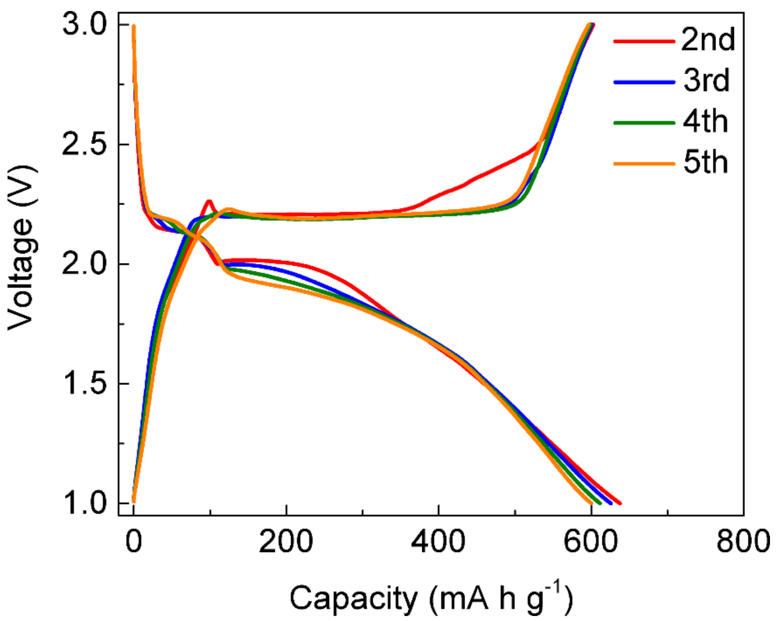

Supplementary Figure 58. Voltage profiles of the Li–Se cell with a $Ti_{0.87}O_2$/PP separator at 0.2C.

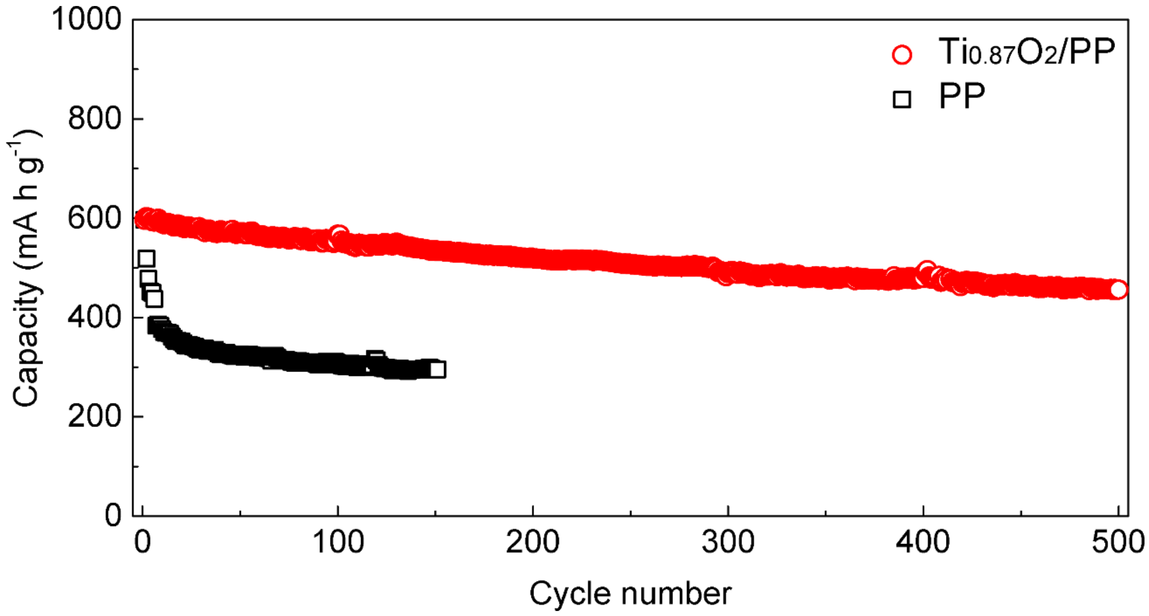

Supplementary Figure 59. Cycling performance of the Li-Se cells at 0.2C with PP and $Ti_{0.87}O_2$/PP separators.

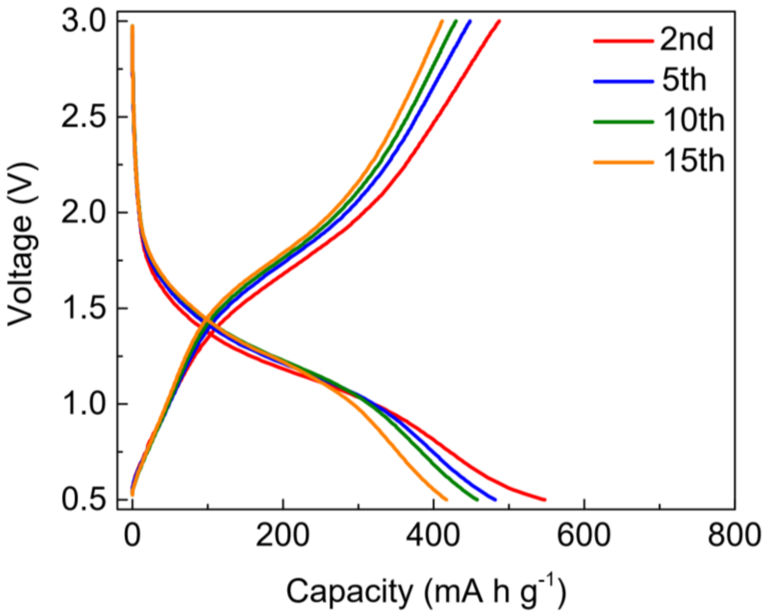

Supplementary Figure 60. Voltage profiles of the Na–Se cell with a PP separator at 0.2C.

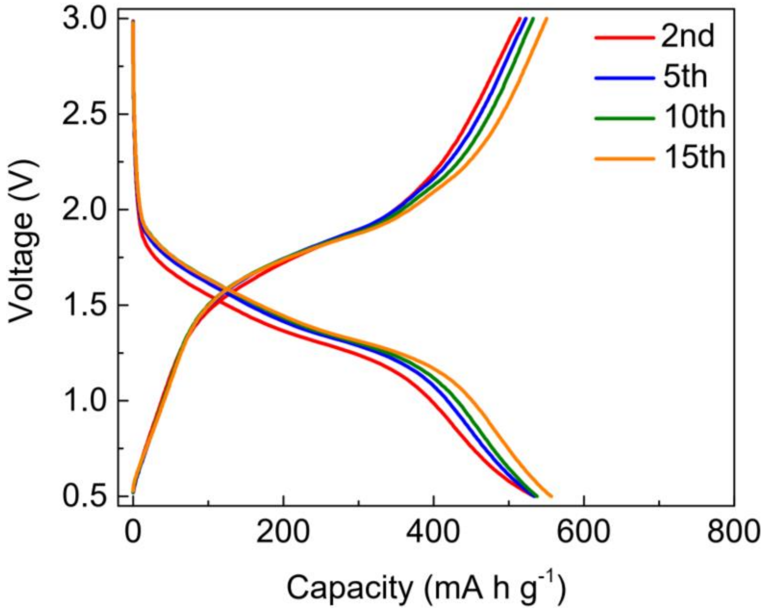

Supplementary Figure 61. Voltage profiles of the Na–Se cell with a $Ti_{0.87}O_2$/PP separator at 0.2C.

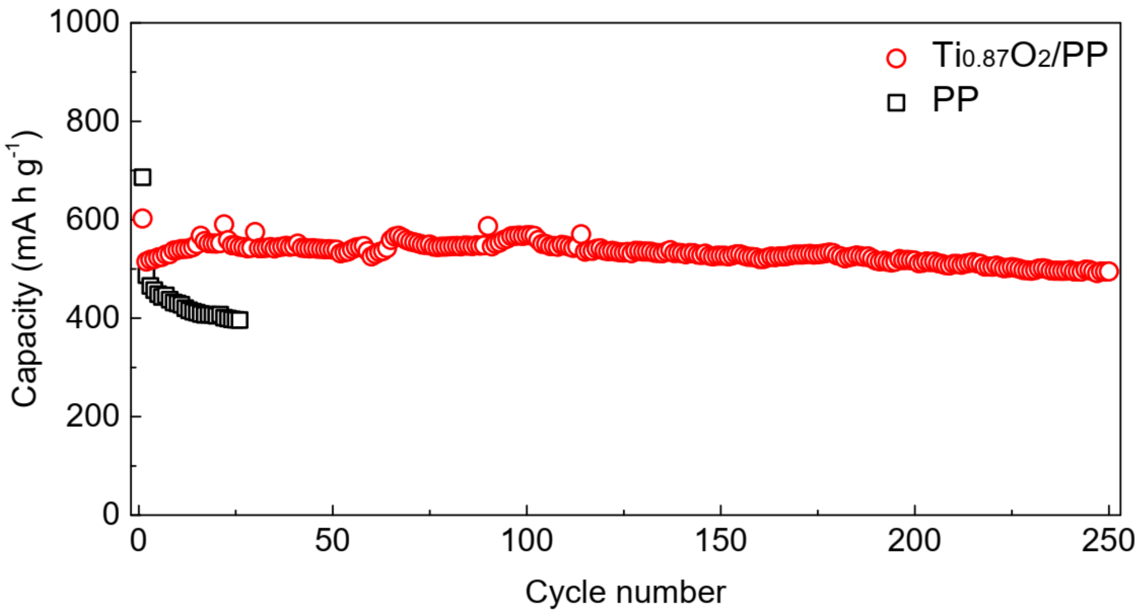

Supplementary Figure 62. Cycling performance of the Na-Se cells at 0.2C with PP and $Ti_{0.87}O_2$/PP separators.

Supplementary Movie 1. Molecular dynamic simulation of the diffusion of polysulfide anions and Li ions through the anionic $Ti_{0.87}O_2$ monolayer with one Ti vacancy.

Supplementary Table 1. Electrochemical properties of various functional separators in Li-S cells.

**Functional separators**			**Battery performance**					**Ref**
**Materials**	**Surface area mass loading (mg cm^{-2})**	**Thickness (μm)**	**Cathode composite active material**	**S wt.% (mg cm^{-2})**	**Voltage range (V)**	**Electrolyte**	**Cycling performance (cycles, C-rates (1C= 1675 mA g^{-1}), capacity decay rate)**	
GO	0.12	5	Carbon black/S	1.0-1.5	1.5-3.0	1 M LiTFSI in DOL/DME (v/v = 1:1)	100, 0.1C, 0.23%	1
Nafion/GO	0.128	0.030	Garphene/ CNT/S	1.2	1.5-3.0	1 M LiTFSI in DOL/DME (v/v = 1:1)	200, 0.1C, 0.18%	2
Commercial graphene	1.3	30	Carbon black/S	1.5-2.1	1.5-2.8	1 M LiTFSI in DOL/DME (v/v = 1:1) with 1.0 wt% $LiNO_3$	500, 0.9C, 0.064%	3
CVD-derived porous graphene	0.54	10	CNT/S	1.8–2.0	1.8-2.8	1 M LiTFSI in DOL/DME (v/v = 1:1) with 1.0 wt% $LiNO_3$	150, 0.5C, 0.16%	4
Commercial graphene@ porous carbon (G@PC)	0.075	0.9	Carbon black/S	3.5	1.6-2.8	1 M LiTFSI in DOL/DME (v/v = 1:1) with 2.0 wt% $LiNO_3$	100, 0.2C, 0.08%	5

Co/N-carbon sheets/reduced graphene oxide	0.2	41.3	CNT/S	1.0	1.7-2.8	1 M LiTFSI in DOL/DME (v/v = 1:1) with 1.0 wt% $LiNO_3$	500, 0.2C, 0.07%	6
Cellular CVD-derived graphene framework	0.3	30	CNT/S	1.2	1.7-2.8	1 M LiTFSI in DOL/DME (v/v = 1:1)	300, 0.8375C, 0.085%	7
B-rGO	0.2-0.3	25	CNT/S	1.45-1.56	1.8-2.8	1 M LiTFSI in DOL/DME (v/v = 1:1) with 0.2 M $LiNO_3$	300, 0.1C, 0.1532%	8
rGO@sodium lignosulfonate (rGO@SL)	0.2	~20	Carbon black/S	1.5	1.7-2.7	1 M LiTFSI in DOL/DME (v/v = 1:1) with 1.0 wt% $LiNO_3$	1000, 2C, 0.026%	9
CNTs/N-doped carbon quantum dot (CNT/NCQD)	0.15	25~30	Carbon black/S	1.3-1.5	1.8-2.7	1 M LiTFSI in DOL/DME (v/v = 1:1) with 2.0 wt% $LiNO_3$	1000, 0.5C, 0.05%	10
CNF-Gum Arabic	0.25	19	CNF/S	1.1	1.7-2.8	1 M LiTFSI in DOL/DME (v/v = 1:1)	250, 1C, 0.024%	11

						with 2.0 wt% $LiNO_3$		
Mg_2Al-LDH	0.018	0.02-0.03	Carbon black/S	1.2-1.4	1.7-2.8	1 M LiTFSI in DOL/DME (v/v = 1:1) with 1.0 wt% $LiNO_3$	200, 0.5C, 0.18%	12
NiFe-LDH/CVD-derived N-doped graphene	0.3	1.5	Carbon/S	1.2	1.7-2.8	1 M LiTFSI in 2.5 M Li_2S_8/tetraglyme	1000, 2C, 0.06%	13
MoS_2	-	0.350	Carbon black/S	-	1.5-3.0	1 M LiTFSI in DOL/DME (v/v = 1:1) with 1.0 wt% $LiNO_3$	600, 0.5C, 0.083%	14
MoS_2-PDDA/PAA	0.1	3	Carbon black/S	1.2-4.0	1.7-2.6	1 M LiTFSI in DOL/DME (v/v = 1:1) with 1.0 wt% $LiNO_3$	2000, 1C, 0.029%	15
Co_9S_8	0.16	-	Carbon black/S	2.0	1.8-2.8	1.85 M $LiCF_3SO_3$ in DOL/DME (v/v = 1:1) with 0.1 M $LiNO_3$	1000, 1C, 0.039%	16

Sb_2Se_{3-x}/rGO	0.5	32	Carbon black/S	1.8	1.7-2.8	1 M LiTFSI in DOL/DME (v/v = 1:1) with 1.0 wt% $LiNO_3$	500, 1C, 0.027%	17
MoP/rGO	0.35-0.45	10	Carbon/S	3.6-4.0	1.8-2.8	0.6 M LiTFSI in DOL/DME (v/v = 1:1) with 0.4 M $LiNO_3$	120, 0.1C, 0.045%	18
Ti_3C_2 MXene	0.1	0.522	Carbon black/S	1.2	1.7-2.8	1 M LiTFSI in DOL/DME (v/v = 1:1) with 0.1 M $LiNO_3$	500, 0.5C 0.062%	19
Black Phosphorus	0.4	~0.35	Carbon black/S	1.5–2	1.7-2.6	1 M LiTFSI in DOL/DME (v/v = 1:1) with 1.0 wt% $LiNO_3$	100, 0.2C, 0.14%	20
Super P/Red phosphorus	0.3	8	Carbon black/S	2	1.5-3.0	1 M LiTFSI in DOL/DME (v/v = 1:1) with 0.1 M $LiNO_3$	500, 1C, 0.036%	21
BN-carbon	-	6~7	Carbon black/S	2.1	1.5-3.0	1 M $LiPF_6$ in EC/DEC (v/v = 1:1)	250, 0.5C, 0.0936%	22

$BaTiO_3$	2.4	18-23	Carbon black/S	3.2	1.8-2.6	1 M LiTFSI in DOL/DME (v/v = 1:1) with 0.3 M $LiNO_3$	50, 0.1C, 0.34%	23
H_xMnO_{2+x}/liquid phase-exfoliated graphene/CNTs	0.2	3	CNT/S	1.8	1.7-2.8	1 M LiTFSI in DOL/DME (v/v = 1:1) with 1.0 wt% $LiNO_3$	1000, 1C, 0.04%	24
TiO_2/commercial graphene	0.15	3	CNT/S	1.2	1.8-2.8	1 M LiTFSI in DOL/DME (v/v = 1:1) with 1.0 wt% $LiNO_3$	300, 0.5C, 0.01%	25
$Li_4Ti_5O_{12}$/chemically exfoliated graphene	0.346	35	Carbon black/S	1.0-1.2	1.7-2.8	1 M LiTFSI in DOL/DME (v/v = 1:1) with 1.0 wt% $LiNO_3$	500, 1C, 0.028%	26
$Ni_3(HITP)_2$	0.066	0.34	CNT/S	8	1.7-2.8	1 M LiTFSI in DOL/DME (v/v = 1:1) with 2.0 wt% $LiNO_3$	500, 1C, 0.066%	27
$Cu_2(CuTCPP)$ nanosheets	0.1	0.5	Carbon black/S	2	1.7-2.8	1 M LiTFSI in DOL/DME (v/v = 1:1) with 2.0 wt%	900, 1C, 0.032%	28

						$LiNO_3$		
CNT@ZIF-8	0.9	15	Carbon black/S	1.2	1.5-3.0	1 M LiTFSI in DOL/DME (v/v = 1:1) with 0.2 M $LiNO_3$	100, 0.2C, 0.45%	29
Ce-MOF/CNT	0.4	8	Carbon black/S	2.5	1.7-2.8	1 M LiTFSI in DOL/DME (v/v = 1:1) with 0.1 M $LiNO_3$	800, 1C, 0.022%	30
MOF@PVDF-HFP	None	28	Carbon cloth/S	1-1.5	1.5-3.0	1 M LiTFSI in DOL/DME (v/v = 1:1) with 0.1 M $LiNO_3$	600, 0.5C, 0.0549%	31
Bacterial cellulose/2D MOF-Co (BC/2D MOF-Co)	2.53	25	Carbon black/S	1.5	1.7-2.8	1 M LiTFSI in DOL/DME (v/v = 1:1) with 1.0 wt% $LiNO_3$	600, 1C, 0.07%	32
MOF@GO	0.3	~10	CMK3/S	0.6-0.8	1.5-3.0	1 M LiTFSI in DOL/DME (v/v = 1:1) with 0.1 M $LiNO_3$	1500, 1C, 0.019%	33

Laponite nanosheets	0.7	3.5	Carbon black/S	1.0-1.2	1.7-2.8	1 M LiTFSI in DOL/DME (v/v = 1:1) with 0.2 M $LiNO_3$	500, 0.2C, 0.06%	34
$Ti_{0.87}O_2$ nanosheets	0.016	0.080	Carbon black/S	1.5<br>3.5	1.7-2.8	1 M LiTFSI in DOL/DME (v/v = 1:1) with 1.0 wt% $LiNO_3$	5000, 1C, 0.0036%<br>4900, 1C, 0.0035%<br>10000, 2C, 0.0035%	This work

Supplementary Table 2. Comparison of Li^+ conductivities of pristine and modified separators.

**Modified separator**	**Li^+ conductivity mS cm^{-1}**	**Pristine separator**	**Li^+ conductivity mS cm^{-1}**	**Ref**
MoS_2/Celgard	0.20	Celgard	0.33	14
LNS/CB-Celgard	0.590	Celgard	0.559	34
MOF@PVDF-HFP	0.094	Celgard	0.138	31
MoS_2-PDDA/PAA	0.48	Celgard	0.51	15
Co-N_x@NPC/G-PP	0.684	PP	0.403	6
$Ti_{0.87}O_2$/PP	0.381 ± 0.028	PP	0.305 ± 0.015	This work

References

[1] Huang, J.-Q. *et al.* Permselective graphene oxide membrane for highly stable and anti-self-discharge lithium-sulfur batteries. *ACS Nano* **9**, 3002–3011 (2015).

[2] Zhuang, T.-Z. *et al.* Rational integration of polypropylene/graphene oxide/nafion as ternary-layered separator to retard the shuttle of polysulfides for lithium-sulfur batteries. *Small* **12**, 381–389 (2016).

[3] Zhou, G. *et al.* A flexible sulfur-graphene-polypropylene separator integrated electrode for ddvanced Li-S batteries. *Adv. Mater.* **27**, 641–647 (2015).

[4] Zhai, P.-Y. *et al.* Scaled-up fabrication of porous-graphene-modified separators for high-capacity lithium-sulfur batteries. *Energy Storage Mater.* **7**, 56–63 (2017).

[5] Pei, F. *et al.* A two-dimensional porous carbon-modified separator for high-energy-density Li-S batteries. *Joule* **2**, 323–336 (2018).

[6] Cheng, Z., Pan, H., Chen, J., Meng, X. & Wang, R. Separator modified by cobalt-embedded carbon nanosheets enabling chemisorption and catalytic effects of polysulfides for high-energy-density lithium-sulfur batteries. *Adv. Energy Mater.* **9**, 1901609 (2019).

[7] Peng, H.-J. *et al.* Janus separator of polypropylene-supported cellular graphene framework for sulfur cathodes with high utilization in lithium-sulfur batteries. *Adv. Sci.* **3**, 1500268 (2016).

[8] Wu, F. *et al.* Light-weight functional layer on a separator as a polysulfide immobilizer to enhance cycling stability for lithium-sulfur batteries. *J. Mater. Chem. A* **4**, 17033–17041 (2016).

[9] Lei, T. *et al.* Inhibiting polysulfide shuttling with a graphene composite separator for highly robust lithium-sulfur batteries. *Joule* **2**, 2091–2104 (2018).

[10] Pang, Y., Wei, J., Wang, Y. & Xia, Y. Synergetic protective effect of the ultralight MWCNTs/NCQDs modified separator for highly stable lithium-sulfur batteries. *Adv. Energy Mater.* **8**,

1702288 (2018).

[11] Tu, S. *et al.* A polysulfide-immobilizing polymer retards the shuttling of polysulfide intermediates in lithium-sulfur batteries. *Adv. Mater.* **30**, 1804581 (2018).

[12] Zhou, Y. *et al.* Cationic two-dimensional sheets for an ultralight electrostatic polysulfide trap toward high-performance lithium-sulfur batteries. *Energy Storage Mater.* **9** 39–46 (2017).

[13] Peng, H.-J. *et al.* A Cooperative interface for highly efficient lithium-sulfur batteries. *Adv. Mater.* **28**, 9551–9558 (2016).

[14] Ghazi, Z. A. *et al.* MoS_2/Celgard separator as efficient polysulfide barrier for long-life lithium-sulfur batteries. *Adv. Mater.* **29**, 1606817 (2017).

[15] Wu, J. *et al.* Ultralight layer-by-layer self-assembled MoS_2-polymer modified separator for simultaneously trapping polysulfides and suppressing lithium dendrites. *Adv. Energy Mater.* **8**, 1802430 (2018).

[16] He, J., Chen, Y. & Manthiram, A. Vertical Co_9S_8 hollow nanowall arrays grown on a Celgard separator as a multifunctional polysulfide barrier for high-performance Li-S batteries. *Energy Environ. Sci.* **11**, 2560–2568 (2018).

[17] Tian, Y. *et al.* Low-bandgap se-deficient antimony selenide as a multifunctional polysulfide barrier toward high-performance lithium-sulfur batteries. *Adv. Mater.* **32**, 1904876 (2020).

[18] Li, M. *et al.* A separator-based lithium polysulfide recirculator for high-loading and high-performance Li-S batteries. *J. Mater. Chem. A* **6**, 5862–5869 (2018).

[19] Song, J. *et al.* Immobilizing polysulfides with MXene-functionalized separators for stable lithium-sulfur batteries. *ACS Appl. Mater. Interfaces* **8**, 29427–29433 (2016).

[20] Sun, J. *et al.* Entrapment of polysulfides by a black-phosphorus-modified separator for lithium-

sulfur batteries. *Adv. Mater.* **28**, 9797–9803 (2016).

[21] Wang, Z. *et al.* Constructing metal-free and cost-effective multifunctional separator for high-performance lithium-sulfur batteries. *Nano Energy* **59**, 390–398 (2019).

[22] Kim, P. J. H. *et al.* Synergistic protective effect of a BN-carbon separator for highly stable lithium sulfur batteries. *NPG Asia Mater.* **9**, e375 (2017).

[23] Yim, T. *et al.* Effective polysulfide rejection by dipole-aligned $BaTiO_3$ coated separator in lithium–sulfur batteries. *Adv. Funct. Mater.* **26**, 7817–7823 (2016).

[24] Lu, Q. *et al.* An "electronegative" bifunctional coating layer: simultaneous regulation of polysulfide and Li-ion adsorption sites for long-cycling and "dendrite-free" Li-S batteries. *J. Mater. Chem. A* **7**, 22463–22474 (2019).

[25] Xiao, Z. *et al.* A lightweight TiO_2/graphene interlayer, applied as a highly effective polysulfide absorbent for fast, long-life lithium-sulfur batteries. *Adv. Mater.* **27**, 2891–2898 (2015).

[26] Zhao Y. *et al.* Dense coating of $Li_4Ti_5O_{12}$ and graphene mixture on the separator to produce long cycle life of lithium-sulfur battery. *Nano Energy* **30**, 1–8 (2016).

[27] Zang, Y. *et al.* Large-area preparation of crack-free crystalline microporous conductive membrane to upgrade high energy lithium-sulfur batteries. *Adv. Energy Mater.* **8**, 1802052 (2018).

[28] Tian, M. *et al.* Ultrathin MOF nanosheet assembled highly oriented microporous membrane as an interlayer for lithium-sulfur batteries. *Energy Storage Mater.* **21**, 14–21 (2019).

[29] Wu, F. *et al.* Metal-organic frameworks composites threaded on the CNT knitted separator for suppressing the shuttle effect of lithium sulfur batteries. *Energy Storage Mater.* **14**, 383–391 (2018).

[30] Hong, X.-J. *et al.* Cerium based metal-organic frameworks as an efficient separator coating catalyzing the conversion of polysulfides for high performance lithium–sulfur batteries. *ACS Nano* **13**,

1923–1931 (2019).

[31] He, Y. *et al.* Simultaneously inhibiting lithium dendrites growth and polysulfides shuttle by a flexible MOF-based membrane in Li-S batteries. *Adv. Energy Mater.* **8**, 1802130 (2018).

[32] Li, Y. *et al.* Single atom array mimic on ultrathin MOF nanosheets boosts the safety and life of lithium-sulfur batteries. *Adv. Mater.* **32**, 1906722 (2020).

[33] Bai, S., Liu, X., Zhu, K., Wu, S. & Zhou, H. Metal-organic framework-based separator for lithium-sulfur batteries. *Nat. Energy* **1**, 16094 (2016).

[34] Yang, Y. & Zhang, J. Highly stable lithium-sulfur batteries based on laponite nanosheet-coated celgard separators. *Adv. Energy Mater.* **8**, 1801778 (2018).
