# Supplementary material for: Atomic-scale regulation of anionic and cationic migration in alkali metal batteries
Source: Nat Commun. 2021 Jul 7;12:4184. doi: 10.1038/s41467-021-24399-9 (PMC8263716; doi:10.1038/s41467-021-24399-9)
Supplement: Supplementary file 3 — Description of Additional Supplementary Files [file 41467_2021_24399_MOESM3_ESM.docx]

**Description of Additional Supplementary Files**

**Supplementary Movie 1.** Molecular dynamic simulation of the diffusion of polysulfide anions and Li ions through the anionic Ti0.87O2 monolayer with one Ti vacancy.
